# Facet‐Controlled Growth of Molybdenum Phosphide Single Crystals for Efficient Hydrogen Peroxide Synthesis

**DOI:** 10.1002/adma.202500250

**Published:** 2025-05-28

**Authors:** Seo Hyun Kim, Jeong‐Hyun Kim, Bogeun Park, Hanhwi Jang, Jeong‐Gyu Lee, Soonmin Yim, Jae Won Jeong, Seyoung Koo, Yeon Sik Jung, Byung‐Hyun Kim, Min‐Jae Choi, Hyeuk Jin Han

**Affiliations:** ^1^ Department of Materials Science and Engineering Sungshin Women's University Seoul 01133 South Korea; ^2^ Department of Department of Advanced Battery Convergence Engineering Dongguk University Pildong‐ro 1‐gil, Jung‐gu Seoul 04620 Republic of Korea; ^3^ Department of Chemical and Molecular Engineering Department of Applied Chemistry Center for Bionano Intelligence Education and Research Hanyang University ERICA 55 Hanyangdaehak‐ro, Sangnok‐gu Ansan‐si Gyeonggi‐do 15588 Republic of Korea; ^4^ Department of Energy and Bio Sciences Department of Applied Chemistry Center for Bionano Intelligence Education and Research Hanyang University ERICA 55 Hanyangdaehak‐ro, Sangnok‐gu Ansan‐si Gyeonggi‐do 15588 Republic of Korea; ^5^ Department of Materials Science and Engineering Korea Advanced Institute of Science and Technology 291 Daehak‐Ro, Yuseong‐Gu Daejeon 34141 Republic of Korea; ^6^ Department of Chemical and Biochemical Engineering Dongguk University Pildong‐ro 1‐gil, Jung‐gu Seoul 04620 Republic of Korea; ^7^ Thin Film Materials Research Center Korea Research Institute of Chemical Technology Daejeon 34114 Republic of Korea; ^8^ Metal Powder Department Korea Institute of Materials Science Changwon 51508 Republic of Korea; ^9^ Department of Energy and Materials Engineering Dongguk University Pildong‐ro 1‐gil, Jung‐gu Seoul 04620 Republic of Korea

**Keywords:** crystal growth, electrocatalysis, facet engineering, molybdenum phosphide, transition metal phosphides

## Abstract

Transition metal phosphides (TMPs) stand out for their excellent catalytic activity, driven by metal‒phosphorus bonding that promotes electron donation, which makes them ideal for electrocatalysis applications. However, the synthesis of single‐crystal TMP, which is essential for elucidating intrinsic properties, remains challenging owing to the lack of efficient methods, low yields, and lengthy processes. This study presents the synthesis of facet‐controlled molybdenum phosphide (MoP) single crystals using a liquid‐metal‐assisted chemical vapor deposition method. By adjusting the synthesis temperature, two distinct MoP morphologies are created: nanoplates dominated by (0001) facets and pillars dominated by $\left(10\bar{1}0\right)$ facets. Electrochemical evaluation reveals that the MoP pillars outperform nanoplates in the two‐electron oxygen reduction reaction, achieving over 92% selectivity for H_2_O_2_ production and significantly higher kinetic current density. Long‐term stability tests confirm that the MoP pillars maintain a high Faradaic efficiency (>90%) and stable electrosynthesis over 80 h of continuous operation, highlighting their robustness. Density functional theory calculations reveal that the $\left(10\bar{1}0\right)$ facets of the pillars enhance catalytic activity by reducing the OOH adsorption strength, thereby lowering the overpotential. This study underscores the importance of facet engineering in optimizing catalytic performance and provides a pathway for designing advanced TMP‐based materials for energy and environmental applications.

## Graphical abstract

Facet‐controlled growth of molybdenum phosphide single crystals via liquid‐metal‐assisted chemical vapor deposition, demonstrating MoP nanoplate and pillar morphologies formed at different temperatures for efficient hydrogen peroxide synthesis.

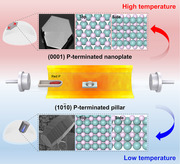

## 1 Introduction

Transition metal phosphides (TMPs) have garnered considerable attention owing to their novel properties, which make them highly versatile for use in various contexts such as batteries,^[^ ^1^ ^]^ low‐dissipation interconnects,^[^ ^2^, ^3^, ^4^, ^5^ ^]^ and catalysts.^[^ ^6^, ^7^, ^8^ ^]^ Within the TMP family, molybdenum phosphide (MoP) stands out for its exotic properties, including high thermal and electrical conductivity,^[^ ^9^, ^10^, ^11^ ^]^ as well as its role as an efficient and cost‐effective catalyst for the hydrogen evolution reaction, which involves a two‐electron transfer process.^[^ ^12^, ^13^, ^14^ ^]^ MoP also demonstrates excellent catalytic activity and stability in various electrolytic environments. These distinctive characteristics position MoP as a promising material for advanced technologies, particularly in energy‐related catalysis and high‐performance electronics.

To fully harness the potential of MoP, single crystals are indispensable as they allow assessments of material characteristics without interference from grain boundaries or defects. However, synthesizing high‐quality MoP single crystals remains challenging owing to the limitations of conventional methods. Traditional approaches for MoP synthesis, such as ion‐mediated synthesis^[^ ^15^, ^16^ ^]^ and the sol‒gel method,^[^ ^17^, ^18^ ^]^ predominantly yield polycrystalline structures. Meanwhile, other techniques, such as chemical vapor transport (CVT) and chemical vapor deposition (CVD), are hindered by slow growth rates and limited yields, rendering them impractical for broader applications. Although CVT has successfully produced MoP single crystals, its inefficiency highlights the need for innovative synthesis methods to enhance yield, efficiency, and crystallographic facet control.

Recently, liquid‐metal‐assisted synthesis has emerged as a promising alternative owing to the exceptional physical and chemical properties of liquid metals,^[^ ^19^, ^20^ ^]^ which offer notable opportunities for catalysis.^[^ ^21^ ^]^ This method enables the synthesis of high‐entropy alloys, two‐dimensional materials, and single crystals, leveraging the low melting points, high surface tension, and self‐limiting oxide layer formation ability of liquid metals.^[^ ^22^, ^23^, ^24^ ^]^ Consequently, this method demonstrates promise for improving single‐crystal growth, affording notable improvements in yield and growth rate.

In this study, we present a method for synthesizing high‐quality MoP single crystals using CVD with liquid Ga as the growth substrate. Driven by its high surface energy, Mo from Mo foil diffuses toward the Ga surface, engaging in a controlled reaction with phosphorus to form MoP single crystals. This approach achieves efficient single‐crystal growth while allowing the modulation of crystallographic facets for specific applications. For instance, MoP nanoplates with dominant (0001) surfaces are synthesized at high temperatures, while MoP pillars with exposed $\left(10\bar{1}0\right)$ surfaces are obtained at lower temperatures.

Building upon these advancements, we investigate the potential of the synthesized high‐quality MoP single crystals as the two‐electron oxygen reduction reaction (2e^−^ ORR) catalysts for hydrogen peroxide (H_2_O_2_) electrosynthesis. Although noble metal catalysts (e.g., Pt, Pd, Au‐Hg alloys) have shown high catalytic activity for 2e^−^ ORR, their high cost limits scalability.^[^ ^25^, ^26^, ^27^ ^]^ In contrast, TMPs are emerging as promising electrocatalysts for 2e^−^ ORR due to their stable structures and excellent catalytic performance.^[^ ^28^ ^]^ Despite numerous reports on MoP for the hydrogen evolution reaction (HER),^[^ ^12^, ^29^ ^]^ its catalytic potential for 2e^−^ ORR has not been previously explored. MoP shows strong potential for 2e^−^ ORR because the Mo‒phosphorus bonding at the surface can readily promote electron donation to oxygen molecules, thereby enhancing ORR activity compared to its oxide counterparts. Furthermore, the low electrical resistance and excellent oxidation resistance of MoP^[^ ^2^ ^]^ make them highly promising for achieving both high activity and long‐term stability. Consequently, we demonstrate stable H_2_O_2_ electrosynthesis over 80 h, outperforming other types of TMP electrocatalyst.^[^ ^28^ ^]^ Using experimental characterization and density functional theory (DFT) predictions, we elucidate the effects of different crystallographic facets on the catalytic efficiency of MoP for selective H_2_O_2_ generation.

H_2_O_2_ has a substantial global market driven by its essential role in water treatment, pulp and paper bleaching, and electronics manufacturing. However, current industrial production methods, particularly the anthraquinone process, face significant challenges such as high energy consumption, environmental pollution, and complex logistics.^[^ ^30^ ^]^ This study represents the first investigation of high‐quality MoP single crystals with distinct crystal facets as potential two‐electron (2e^−^) ORR catalysts, a promising route for renewable and on‐site H_2_O_2_ generation as an alternative to the anthraquinone process. By presenting an efficient method for synthesizing high‐quality TMP single crystals and clarifying their applications, our research advances material synthesis and catalysis for H_2_O_2_ electrosynthesis.

## 2 Results

### 2.1 Synthesis of Facet‐Controlled MoP Crystals

**Figure 1:**
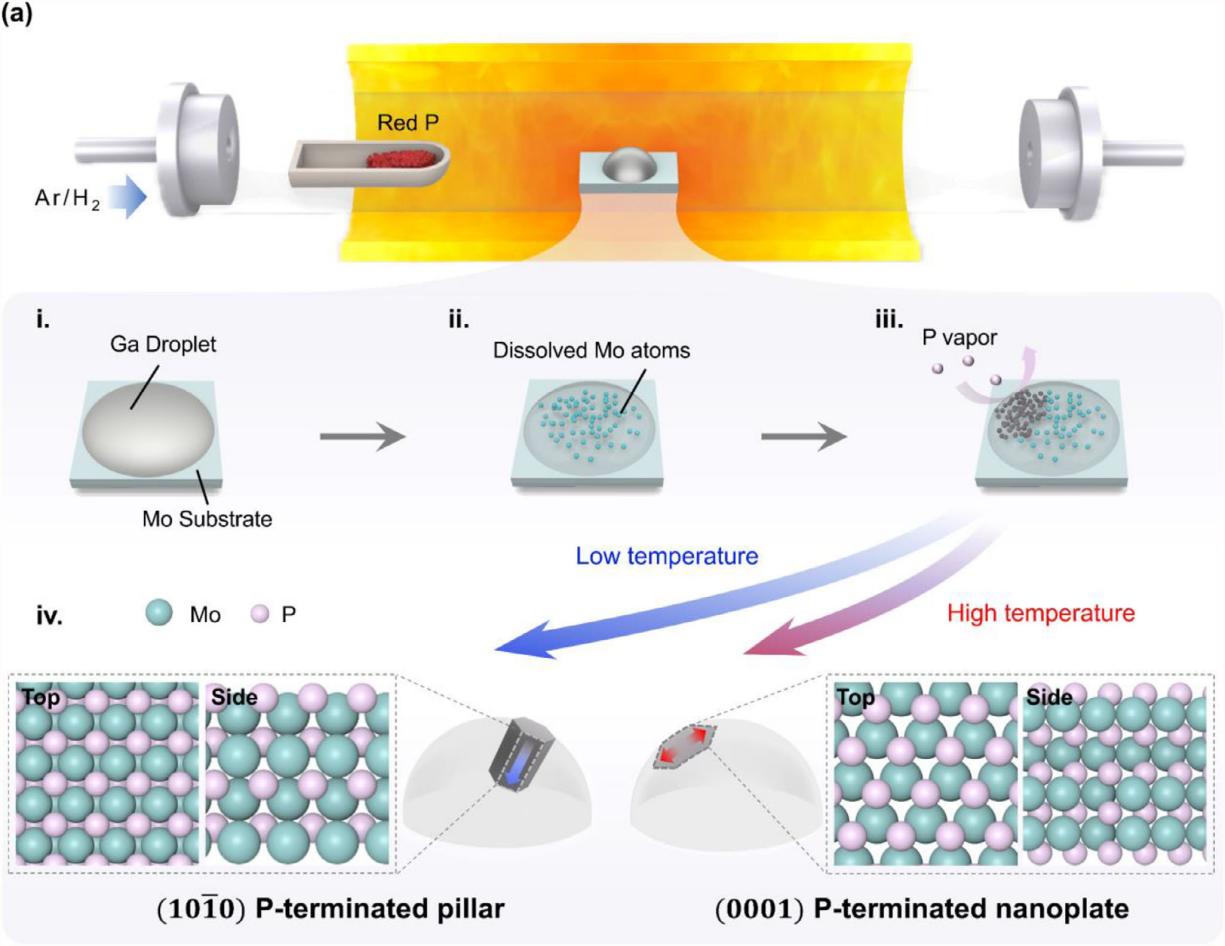
Schematic of MoP single‐crystal synthesis. a) Illustration of the CVD growth process for MoP single crystals using a horizontal tube furnace. b) Mechanistic pathway for MoP single‐crystal formation: (i) Initial deposition of a Ga droplet on the Mo substrate, (ii) diffusion of Mo atoms into the Ga droplet, (iii) introduction of red phosphorus vapor during CVD, and (iv) crystal structures formed depending on synthesis temperature. At high temperatures, MoP nanoplates with (0001) surfaces are formed, illustrated from both top and side views. At low temperatures, MoP pillars with $\left(10\bar{1}0\right)$ surfaces are produced, also illustrated from top and side views. Teal and pink spheres represent Mo and phosphorus atoms, respectively.

High‐quality MoP single crystals were synthesized using a liquid‐metal‐assisted CVD method (**Figure** **1a**). A piece of Mo foil served as both the support for the Ga substrate and the Mo source, while red phosphorus acted as the phosphorus source (Figure 1b(i)). At high growth temperatures, Mo diffuses toward the liquid‐metal Ga surface, driven by its high surface energy, as depicted in Figure 1b(ii).^[^ ^19^ ^]^ Specifically, the low solubility of Mo in Ga promotes efficient extraction from the metal substrate.^[^ ^31^ ^]^ Mo then reacts with phosphorus on the Ga surface, forming MoP nuclei (Figure 1b(iii)). Controlling the growth of specific planes is essential for tailoring the structural and functional characteristics of MoP for various applications. At high temperatures, MoP nanoplates with (0001) surfaces are synthesized, whereas at lower temperatures, MoP pillars with $\left(10\bar{1}0\right)$ surfaces are formed (Figure 1b(iv)).

To control the dominant crystallographic plane of MoP crystals, the calculated surface energies of various planes were examined to theoretically understand the morphologies of the MoP single crystals. Our DFT calculations reveal that under phosphorus‐rich conditions, the P‐terminated (0001) surface exhibits the lowest surface free energy, making it the most thermodynamically stable orientation (Figure S1, Supporting Information).^[^ ^32^ ^]^ Consequently, nucleation is expected to preferentially occur on the (0001) surface.

**Figure 2:**
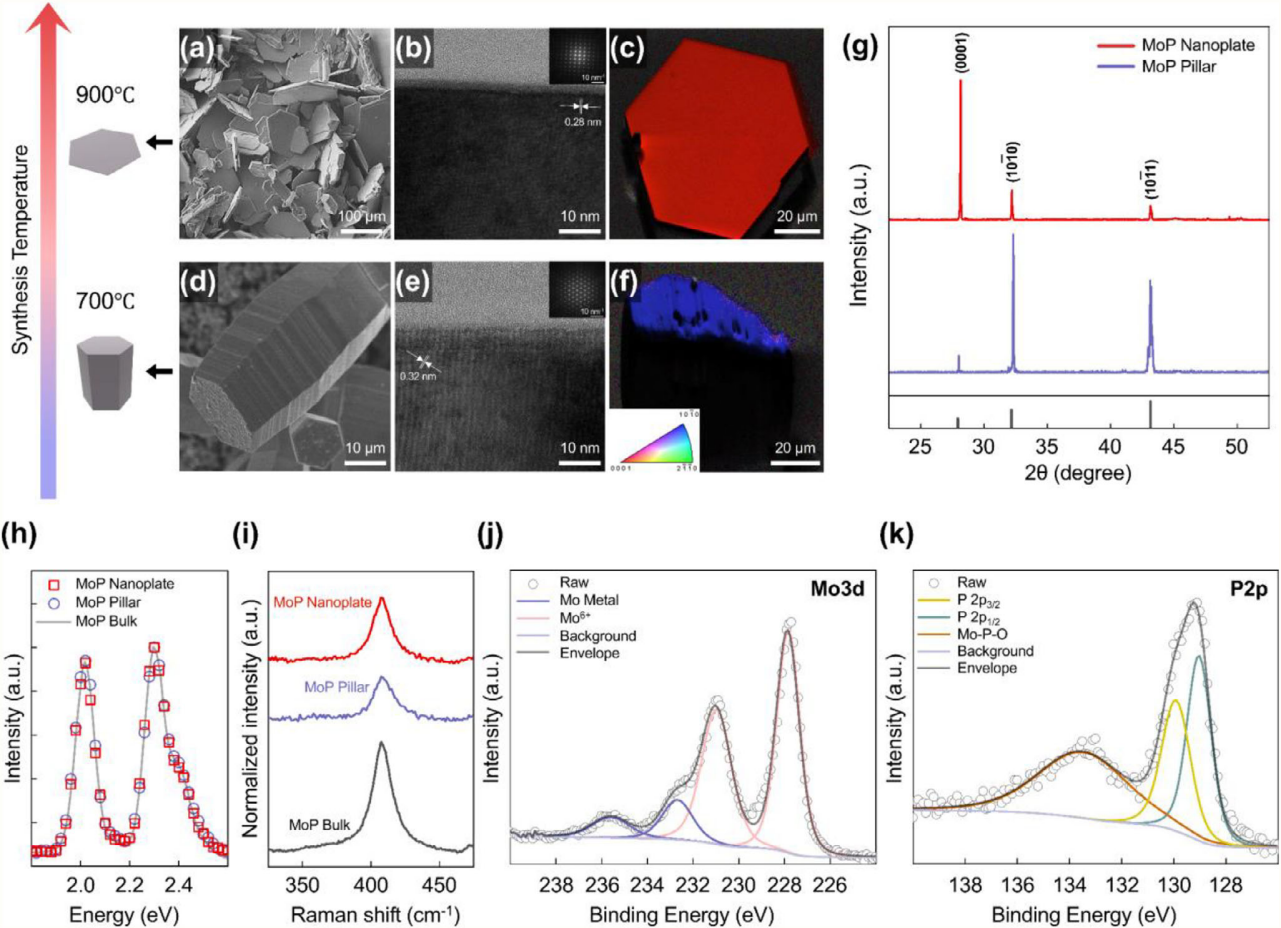
Characterization of MoP single crystals. a) SEM image, b) High‐resolution TEM image of nanoplate cross‐section (Inset in (b) show the SAED pattern), and c) EBSD inverse pole figure‐z image of MoP nanoplates synthesized at 900 °C, highlighting their hexagonal morphology and smooth surfaces. d) SEM image, e) High‐resolution TEM image of pillar cross‐section (Inset in (e) shows the SAED pattern), and f) EBSD inverse pole figure‐z image of MoP pillars synthesized at 700 °C, displaying their elongated structure and $\left(10\bar{1}0\right)$ facet orientation. The inset in (f) displays the inverse pole figure color triangle for crystallographic orientations. g) X‐ray diffraction (XRD) patterns of MoP nanoplates and pillars, confirming their crystallographic orientation and phase. h) Energy dispersive spectroscopy (EDS) spectra of MoP nanoplates and pillars overlapped with that of the reference MoP bulk crystal. i) Raman spectra of MoP nanoplates and pillars and the reference MoP bulk crystal, exhibiting a single identifiable phonon mode. a.u., arbitrary units. j–k) X‐ray photoelectron spectroscopy (XPS) analysis of Mo 3d and P 2p binding energies, validating the chemical composition and bonding environment of MoP nanostructures.

Temperature plays a critical role in determining the balance between thermodynamic stability and kinetic growth rates, ultimately influencing nucleation and growth rates. To investigate this, we assessed the relationship between growth temperature and crystal morphology. Notably, at higher temperatures (900 °C), increased atomic mobility enables lateral diffusion, facilitating the expansion of (0001) facets and leading to nanoplate formation. DFT calculations of Mo adatom diffusion on (0001) reveal a diffusion energy barrier of 2.1 eV, which is readily overcome at higher temperatures due to enhanced surface diffusion (Figure S2, Supporting Information). These structures are observed in SEM images (**Figure** **2**a) and further characterized by transmission electron microscopy (TEM). TEM analysis of nanoplate‐type MoP single crystals revealed a vertical axis oriented along the $\left(10\bar{1}0\right)$ plane (Figure 2b). High‐resolution TEM imaging showed distinct lattice spacings of 0.28 nm, consistent with the $\left(10\bar{1}0\right)$ planes. Selected‐area electron diffraction (SAED) patterns exhibited clear symmetry along the $\left[10\bar{1}0\right]$ zone axis, confirming the single‐crystalline nature. The surface facets of the (0001) plane were verified by electron backscatter diffraction (EBSD) results (Figure 2c).

As the temperature decreases, limited atomic mobility inhibits lateral diffusion, restricting growth along the (0001) plane and instead promoting vertical stacking along 〈0001〉, resulting in a $\left(10\bar{1}0\right)$ surface morphology that resembles a pillar structure, referred to as MoP pillars. At lower temperatures (700 °C), the reduced thermal energy prevents Mo adatoms from overcoming the 2.1 eV diffusion barrier, leading to their accumulation on (0001) rather than diffusing laterally. This results in preferential growth along 〈0001〉, ultimately exposing the $\left(10\bar{1}0\right)$ surface and forming the pillar morphology. This pillar‐like structure is observed in the SEM images (Figure 2d) and further analyzed by detailed TEM characterization. TEM analysis of pillar‐type MoP single crystals revealed a vertical axis orientation along the (0001) plane (Figure 2e). High‐resolution TEM imaging showed lattice spacings of 0.32 nm for the (0001) planes, and SAED patterns exhibited clear hexagonal symmetry along the [0001] zone axis, conclusively confirming their single‐crystalline nature. The surface facets of the $\left(10\bar{1}0\right)$ plane were verified by EBSD results (Figure 2f). Further dimensional characterization showed that nanoplates had average widths of 139.92 µm ± 32.85 and thicknesses of 0.807 µm ± 0.11, whereas pillars showed average widths of 27.3 µm ± 2.64 and thicknesses of 63.50 µm ± 10.70.

The crystal structures of the synthesized MoP samples were confirmed by XRD analysis. Figure 2g illustrates the XRD patterns of the MoP nanoplates and pillars. These XRD patterns display characteristic diffraction peaks of MoP (JCPDS card No. 65–6024), confirming the successful synthesis of MoP crystals. The relative intensities of the (0001) and (1010) diffraction peaks in the XRD patterns of the nanoplates and pillars suggest a preferred growth orientation.

EDS analysis confirmed the elemental composition of the synthesized MoP crystals, with an Mo:P atomic ratio close to 1:1. This result was inferred from the overlap between the EDS spectra of both crystals and that of an MoP bulk crystal grown via CVT (Figure 2h).^[^ ^2^ ^]^ Overall, these experimental findings demonstrate the high purity of MoP. The Raman spectra of both MoP single crystals display a notable peak at 409 cm^−1^, consistent with previous reports (Figure 2i).^[^ ^2^ ^]^ Calculations reveal that the E mode is Raman active owing to the absence of inversion symmetry in the MoP crystals.^[^ ^33^ ^]^ This similarity suggests that the synthesized crystals share structural characteristics with previously characterized MoP materials, validating the effectiveness of not only the growth method but also the facet‐controlling technique.

Additionally, XPS was used to analyze the surface electronic state of each element in the MoP crystals. The Mo 3d spectrum displays peaks at 230.97 and 227.83 eV, corresponding to Mo in MoP (Figure 2j).^[^ ^34^, ^35^ ^]^ Meanwhile, the P 2p spectrum displays two distinct peaks at 129.03 and 129.92 eV, corresponding to the P 2p_3/2_ and P 2p_1/2_ states of phosphorus bonded to Mo (Figure 2k), respectively.^[^ ^2^, ^34^, ^35^ ^]^ Furthermore, the P 2p spectrum exhibits an additional peak at a higher binding energy (≈133.0 eV), attributed to oxidized phosphorus species (PO_4_^3−^),^[^ ^36^ ^]^ indicating a P‐terminated surface, which is critical for catalytic activity. Mo/P ratios of 1.02 ± 0.03 were observed across five analyzed surface regions, indicating homogeneity of active sites. High‐resolution O 1s spectra revealed two distinct contributions: a peak at 532 eV (28% intensity), corresponding to non‐lattice oxygen species (e.g., O_2_^2−^), and a peak at 533 eV (72% intensity), assigned to adsorbed oxygen,^[^ ^37^ ^]^ enhancing hydrophilicity and thus improving reactant accessibility (Figure S3, Supporting Information). Notably, lattice oxygen accounted for less than 1% of the O 1s signal, underscoring the high phase purity of the prepared MoP crystals.

In addition, fitted Ga 2p spectra (Figure S3, Supporting Information) display no Ga–P peak at 1117.3 eV, indicating that phosphorus does not react with gallium. The total Ga impurities are under 2 at%, further confirming the high purity of the prepared MoP crystals.^[^ ^38^ ^]^ ICP‐MS analysis revealed trace Ga impurities of 518.16 ppm, higher than commercial MoP power (16.03 ppm), yet sufficiently low to suggest that further optimization of etching conditions could reduce impurities (Table S1, Supporting Information). Moreover, TEM Energy Dispersive X‐ray Spectroscopy confirmed no detectable Ga within individual crystals (detection limit ≈0.5 at%), confirming that the MoP crystals themselves are intrinsically free of Ga impurities (Figure S4, Supporting Information). Besides the high purity, our synthesis method provides remarkable production efficiency (≈ 60 mg h^−1^, Figure S5, Supporting Information), representing a ≈10 000‐fold enhancement compared to traditional CVT,^[^ ^4^ ^]^ accompanied by effective facet control and rapid growth rates (Table S2, Supporting Information).

### 2.2 Facet‐Dependent Electrochemical Catalysis

**Figure 3:**
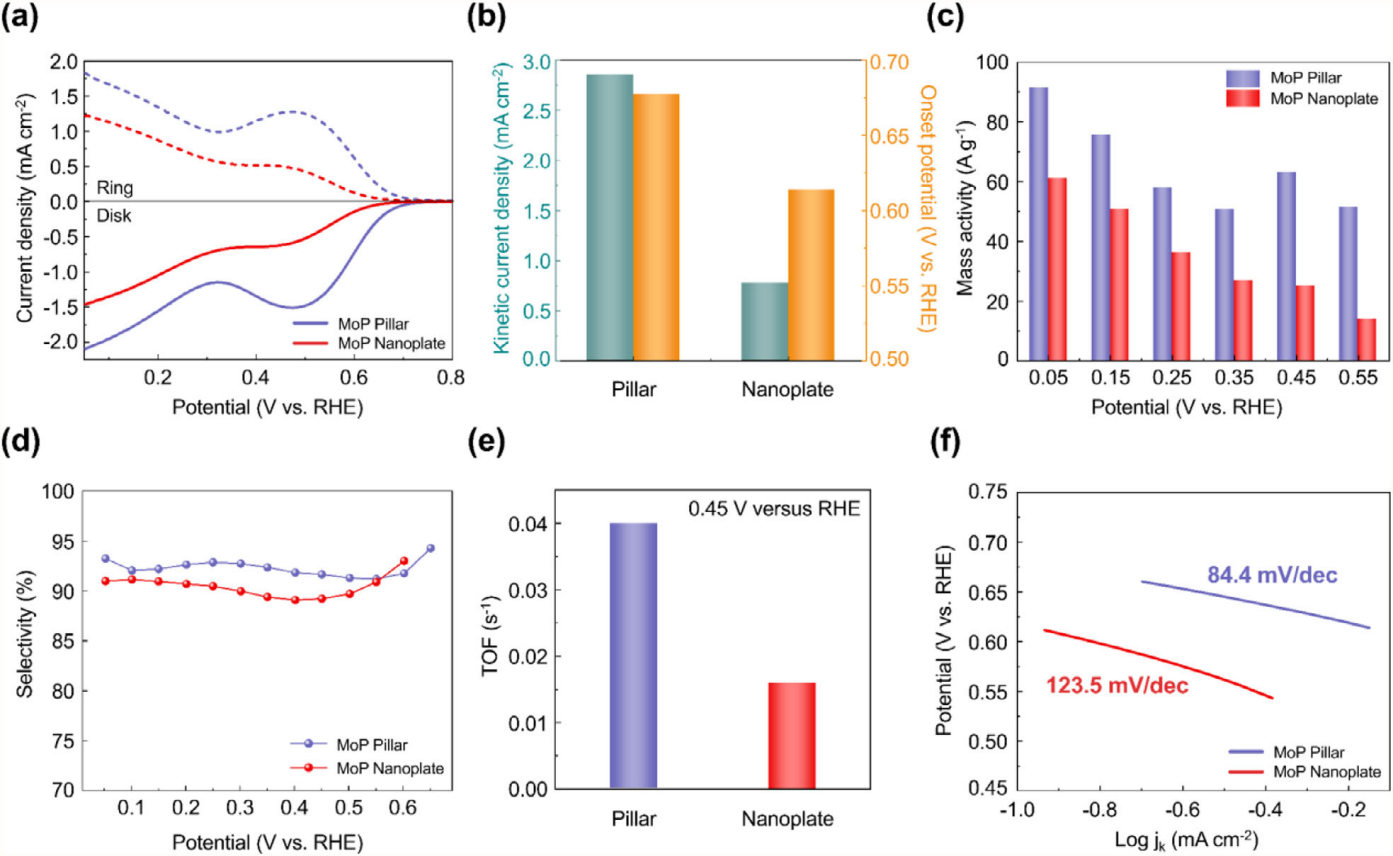
Electrochemical ORR performance for H_2_O_2_ production based on the exposure of different crystal planes of the MoP catalysts. a) Comparison of ORR performance and simultaneous H_2_O_2_ detection current densities at the ring electrode for the MoP pillar and nanoplate catalysts in O_2_‐saturated 0.1 M KOH at a sweep rate of 10 mV s^−1^. H_2_O_2_ currents were estimated from the ring currents and the calculated collection efficiency (Figure S11, Supporting Information). b) Kinetic current density at 0.45 V versus RHE and onset potential at a disk current density of 0.1 mA cm^−2^. c) Mass activity of the 2e^−^ reaction. d) Calculated H_2_O_2_ selectivity. e) TOF. f) Mass‐transfer‐corrected Tafel plots of kinetic current densities for H_2_O_2_ production.

To assess the effects of the crystallographic planes of the synthesized MoP crystals on their electrochemical performance, we performed ORR analysis of the MoP pillars and nanoplates in O_2_‐saturated 0.1 M KOH using a rotating ring‐disk electrode (RRDE). **Figure** **3**a illustrates the corresponding linear sweep voltammetry (LSV) polarization curves. Here, the solid lines represent oxygen reduction currents measured at the disk electrode, indicating the efficiency of the ORR process. Meanwhile, the dashed lines correspond to H_2_O_2_ oxidation currents measured at the ring electrode, reflecting the extent of H_2_O_2_ formation.

The LSV polarization curves indicate that the MoP pillars produced 2.6 times greater H_2_O_2_ currents (1.28 mA cm^−2^ at 0.45 V versus RHE) than the MoP nanoplates (0.49 mA cm^−2^ at 0.45 V versus RHE). To further assess their performance, the kinetic current density for H_2_O_2_ production was calculated by correcting for mass transport limitations using the Koutecky‒Levich equation (see Experimental Section). The calculated kinetic current density for H_2_O_2_ production was 2.85 mA cm^−2^ for the MoP pillars (at 0.45 V versus RHE), significantly higher than that for the MoP nanoplates (0.77 mA cm^−2^, Figure 3b). Moreover, the MoP pillars exhibited a lower ORR overpotential, with an onset potential of 0.68 V (versus RHE), compared to 0.61 V (versus RHE) for the MoP nanoplates. Hence, as illustrated in Figure 3c, the MoP pillars demonstrated significantly higher 2e^−^ ORR mass activity than the MoP nanoplates across the potential range of 0.05‒0.55 V (versus RHE). These ORR results indicate that the MoP pillars selectively generate H_2_O_2_ via the 2e^−^ ORR pathway, achieving over 92% H_2_O_2_ selectivity at 0.45 V versus RHE (Figure 3d). These observations also reveal that the ($10\bar{1}0$) surface exhibits superior electrocatalytic performance than the (0001) surface. The selective exposure of ($10\bar{1}0$) planes in the MoP pillars optimizes the binding strength of *OOH intermediates, lowering the overpotential, enhancing 2e^−^ ORR kinetics, and achieving a higher current density compared to MoP nanoplates. The difference in the catalytic performance of the pillars and nanoplates emphasizes the importance of controlling exposed crystal facets for designing efficient ORR electrocatalysts. To confirm that the MoP retains its structural features after electrode coating, we performed SEM measurements for the MoP nanoplates, and pillars collected from the electrode surface (Figure S6, Supporting Information). The SEM images confirmed that their morphologies were preserved, in agreement with the pristine structure shown in Figure 2a,d.

The turnover frequency (TOF) of the MoP pillars was determined to be 0.04 s⁻^1^, 2.5 times that of the MoP nanoplates (0.016 s⁻^1^) (Figure 3e). This enhanced TOF reflects improved 2e^−^ ORR kinetics, resulting in sustained and efficient H_2_O_2_ production. To further analyze reaction kinetics, Tafel slope measurements were conducted. The results revealed that the MoP pillars exhibited a smaller Tafel slope of 84.4 mV dec⁻^1^ compared to the MoP nanoplates (123.5 mV dec⁻^1^), indicating faster 2e⁻ ORR kinetics (Figure 3f). The electrochemically active surface area (ECSA) of the catalysts was determined using the double‐layer capacitance (C_dl_) method derived from cyclic voltammetry (CV) curves (Figure S7, Supporting Information).^[^ ^39^ ^]^ The obtained C_dl_ values were 9.52 µF cm⁻^2^ for the MoP pillars and 7.04 µF cm⁻^2^ for the MoP nanoplates. ECSA‐normalized LSV curves revealed that the MoP pillars exhibited 1.8 times higher ORR current density compared to the MoP nanoplates (at 0.45 V versus RHE), indicating their enhanced intrinsic catalytic activity.

To evaluate the advantages of single crystal MoP versus polycrystalline MoP, we synthesized polycrystalline MoP as a reference material (see the method on Note S1, Supporting Information).^[^ ^2^, ^40^ ^]^ XRD analysis revealed that the polycrystalline MoP comprises multiple crystal planes, including (0001), $\left(10\bar{1}0\right)$, $\left(10\bar{1}1\right)$ and $\left(2\bar{1}\bar{1}0\right)$ planes (Figure S8, Supporting Information). LSV polarization curves (Figure S9, Supporting Information) revealed that the polycrystalline MoP exhibited a H_2_O_2_ current density of 0.61 mA cm^−2^ at 0.45 V (versus RHE), which is slightly higher than that of the MoP nanoplates but considerably lower than that of the MoP pillars (Figure S9, Supporting Information). Considering their ECSA values (MoP nanoplates: 7.04 µFcm⁻^2^, MoP polycrystalline: 14.2 µF cm⁻^2^), the ECSA‐normalized ORR activity of the polycrystalline MoP was even lower than that of the MoP nanoplates (Figure S10, Supporting Information). These results support the superior catalytic performance of single crystal MoP, which benefits from controlled facets and enhanced electrical conductivity compared to polycrystalline MoP.^[^ ^2^ ^]^

### 2.3 Continuous H_2_O_2_ Electrosynthesis

**Figure 4:**
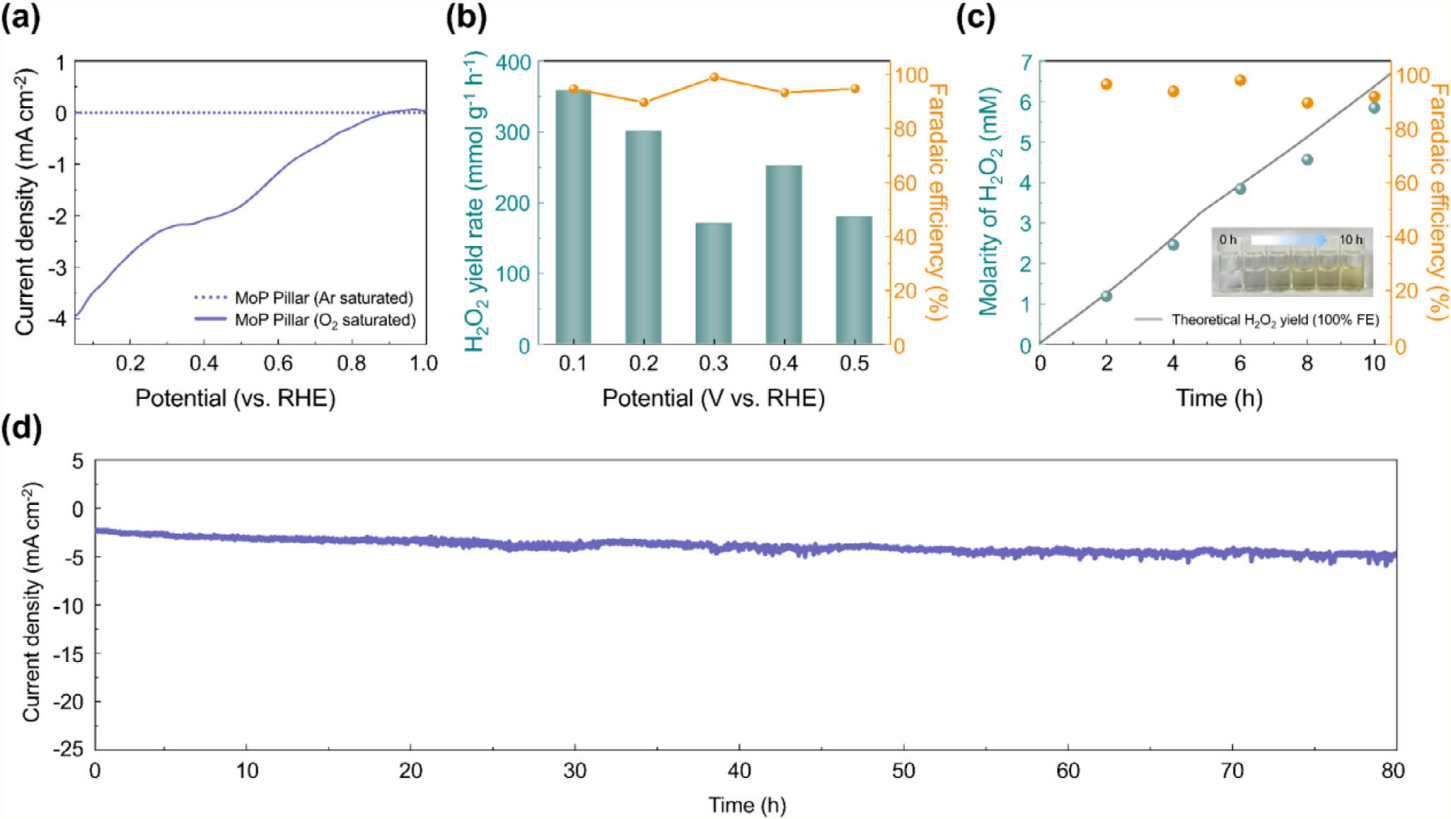
Stability and yield of the H_2_O_2_ electrocatalysts in an H‐Cell. a) LSV curves of the MoP pillars in 0.1 M O_2_‐ and N_2_‐saturated KOH. b) H_2_O_2_ yield rates and FEs of the MoP pillars across voltages ranging from 0.1‒0.5 V. The electrolyte was reacted with a 0.1 M TiOSO4 solution to produce yellow‐colored H_2_TiO_4_. c) Theoretical H_2_O_2_ yield derived from the corresponding chronoamperometry curve (Figure S13, Supporting Information), alongside the detected H_2_O_2_ molarity and calculated FE over 10 h at 0.1 V. The inset image illustrates the color change of the reaction mixture over time (left to right). d) Long‐term chronoamperometry of the MoP pillars at 0.1 V for 80 h.

Electrochemical tests were performed in a three‐electrode setup within a divided H‐type cell to evaluate the potential of the MoP pillar catalysts for continuous H_2_O_2_ electrosynthesis, using O_2_‐ or Ar‐saturated 0.1 M KOH as the electrolyte. The MoP pillar catalysts were coated onto carbon paper at a loading rate of 0.1 mg cm^−2^. A Nafion membrane was used to isolate the working electrode (WE) from the counter electrode (CE), preventing H_2_O_2_ decomposition at the CE. LSV curves revealed that the MoP pillars exhibit negligible currents in the Ar‐saturated electrolyte (dashed line in **Figure** **4**a). Conversely, significant current responses under O_2_‐saturated conditions confirmed the occurrence of the ORR (solid line in Figure 4a).

Next, Faradaic efficiency (FE) and H_2_O_2_ yield rate were determined by quantifying the amount of accumulated H_2_O_2_ in the cathode chamber during the ORR process, using a colorimetric method involving a reaction with a TiOSO_4_ solution (see Experimental Section). After 1 h of operation, an electrolyte sample was extracted and reacted with a 0.1 M TiOSO_4_ solution to quantify H_2_O_2_ production across the potential range of 0.1−0.5 V (Figure S12, Supporting Information). As depicted in Figure 4b, the H_2_O_2_ yield rate over the MoP pillars steadily increased with the overpotential, peaking at 359.97 mmol g^−1^ h^−1^ at 0.1 V (versus RHE). The FE of the 2e^−^ ORR on the MoP pillars remained consistently above 90% across all tested potentials.

We also measured the concentration of accumulated H_2_O_2_ generated by the MoP pillars over 10 h at 0.1 V (versus RHE). The H_2_O_2_ concentration reached 7.6 mM after 10 h, while the FE remained steadily above 90% (Figure 4c). To evaluate the long‐term stability of the MoP pillars, chronoamperometry was performed at a constant potential of 0.1 V versus RHE. The current density remained stable at ≈ −3.5 mA cm^−2^ over 80 h (Figure 4d). This enhanced stability can be attributed to the robust structural integrity of the MoP single crystals and their resistance to phosphide oxidation under ORR conditions. These results strongly support the potential of MoP single crystals as practical and scalable electrocatalysts for sustainable H_2_O_2_ production.

### 2.4 DFT Analysis of the Facet‐Dependent MoP Catalytic Performance for H_2_O_2_ Electrocatalysis

**Figure 5:**
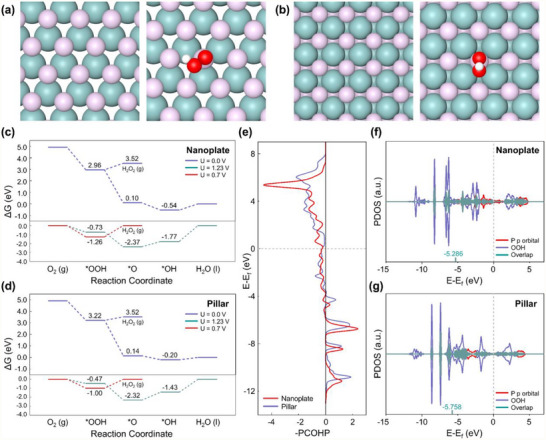
DFT analysis of facet‐dependent catalytic performance of MoP: Atomic configurations of a) bare and OOH‐adsorbed (0001) phosphorus‐terminated nanoplate surfaces and b) bare and OOH‐adsorbed $\left(10\bar{1}0\right)$ phosphorus‐terminated pillar surfaces. Calculated Gibbs free energy diagrams for the 2e^−^ and 4e^−^ pathways on c) the MoP nanoplate and d) MoP pillar. e) COHP analyses of the MoP nanoplate and pillar. PDOS plots for f) the nanoplate surface and g) pillar surface with adsorbed OOH, highlighting overlapping regions.

To complement the experimental observation of the superior H_2_O_2_ production efficiency of MoP pillars compared to that of nanoplates, we performed DFT calculations to investigate whether these performance differences originated from their distinct structural features. The XRD data presented in Figure 2g reveal that the (0001) peak is the most dominant for the nanoplate morphology, while the $\left(10\bar{1}0\right)$ peak is the most prominent for the pillar morphology. As illustrated in Figure 1a, during CVD, the introduction of phosphorus gas leads to MoP formation in a phosphorus‐rich environment. Surface free energy calculations as functions of the chemical potential of phosphorus reveal that the phosphorus‐terminated (0001) and phosphorus‐terminated $\left(10\bar{1}0\right)$ structures are favored under phosphorus‐rich conditions (Figure S1, Supporting Information). While local reconstruction, which could expose metallic atoms in phosphides,^[^ ^28^, ^41^ ^]^ cannot be completely ruled out, our results indicate that P‐terminated configurations remain energetically preferred. Thus, under the synthesis conditions in this study, phosphorus atoms are likely to serve as the primary adsorption sites, whereas less‐exposed Mo atoms play a more limited role in catalysis. These findings are consistent with earlier reports suggesting that phosphide surfaces can expose P atoms depending on specific experimental parameters.^[^ ^42^, ^43^, ^44^ ^]^ Consequently, based on both XRD data and DFT surface free energy calculations, we modeled the nanoplate morphology with a phosphorus‐terminated (0001) surface and the pillar morphology with a phosphorus‐terminated $\left(10\bar{1}0\right)$ surface (**Figure** **5**a,b).

Adsorption sites on each surface were classified as “P_Top,” “PP_Bridge,” and “PPP_Hollow,” based on their atomic configurations. For instance, the site directly above a phosphorus atom was designated as “P_Top,” the site between two phosphorus atoms was labeled as “PP_Bridge,” and the hollow site formed by three phosphorus atoms was called “PPP_Hollow” (Figure S14, Supporting Information). Each site was evaluated for its interaction with adsorbates relevant to the H_2_O_2_ production pathway and the competing ORR.^[^ ^45^, ^46^, ^47^ ^]^ Adsorption calculations revealed that all adsorbates exhibited the strongest binding affinity for the P_Top site (Figure S15, Supporting Information). Gibbs free energy calculations were then performed for H_2_O_2_ production and the ORR to determine the required overpotentials, with the corresponding results illustrated in Figure 5c,d. The overpotentials for H_2_O_2_ production were determined to be 1.26 V for the (0001) surface and 1.00 V for the $\left(10\bar{1}0\right)$ surface, indicating that the $\left(10\bar{1}0\right)$ surface is more favorable for H_2_O_2_ production. This aligns with the experimental results, demonstrating improved H_2_O_2_ production efficiency of the MoP pillars with dominant $\left(10\bar{1}0\right)$ surfaces. Additionally, the overpotential for the 4e^−^ ORR on the $\left(10\bar{1}0\right)$ surface was found to be 1.43 V, significantly higher than that for H_2_O_2_ production. This suggests strong selectivity for the 2e^−^ H_2_O_2_ generation pathway on the $\left(10\bar{1}0\right)$ surface.

As depicted in Figure 5c,d, OOH adsorption is a crucial step in determining the overpotential for H_2_O_2_ production. Notably, the $\left(10\bar{1}0\right)$ surface exhibited weaker OOH adsorption compared to the (0001) surface. To compare the OOH adsorption strength between the nanoplate and pillar morphologies, crystal orbital Hamilton population (COHP) calculations were performed (Figure 5e).^[^ ^48^ ^]^ Our COHP analysis focused on the interaction between the phosphorus atom, the active site on the MoP surface, and the oxygen atom of the OOH molecule. The COHP results revealed a less negative integrated COHP value for the $\left(10\bar{1}0\right)$ surface (−1.753 eV) compared to that for the (0001) surface (−2.019 eV), indicating weaker adsorption on the pillar structure. This reduced adsorption strength lowers the overpotential, favoring H_2_O_2_ production on the $\left(10\bar{1}0\right)$ surface. Additionally, projected density of states (PDOS) calculations was conducted to further analyze the binding strength, supporting the COHP findings (Figure S16, Supporting Information). This PDOS analysis also focused on the surface phosphorus atom and the oxygen atom of OOH (Figure 5f,g). Overlapping regions in the PDOS, highlighted in green, represent shared electron density between the atoms. The center of the overlapping band is located at −5.286 eV for the MoP nanoplate and −5.758 eV for the MoP pillar. The deeper band center for the $\left(10\bar{1}0\right)$ surface indicates weaker binding interactions, correlating with its lower overpotential.

Our DFT calculations reveal that the superior H_2_O_2_ production performance of the MoP pillar morphology stems from its predominantly exposed $\left(10\bar{1}0\right)$ surface, which exhibits weaker OOH adsorption strength closer to the optimal value. This reduced adsorption strength results in a lower overpotential compared to that for the (0001) surface of the nanoplate These findings corroborate the experimental observations and provide atomic‐scale insights into the structure‒performance relationship of MoP catalysts. These insights are expected to be instrumental in designing advanced electrocatalysts for efficient H_2_O_2_ production.

## 3 Conclusion

In summary, this study demonstrated the growth of high‐quality, facet‐controlled MoP single crystals using a liquid‐metal‐assisted CVD process. By systematically varying the synthesis temperature, we achieved control over crystal morphologies through reaction rate modulation on the surface, resulting in two distinct structures: MoP nanoplates dominated by (0001) facets and MoP pillars with exposed $\left(10\bar{1}0\right)$ facets. Electrochemical evaluations of the MoP nanoplates and pillars revealed the notable influence of crystal facets on the selectivity and activity of the catalysts. For instance, the MoP pillars, characterized by exposed $\left(10\bar{1}0\right)$ facets, demonstrated superior selectivity and activity for the 2e^−^ ORR pathway, achieving over 92% H_2_O_2_ selectivity and a high kinetic current density. The lower overpotential and enhanced TOF of the MoP pillars corresponded to their optimal binding strength for *OOH intermediates, facilitating a more efficient reaction mechanism, as corroborated by DFT calculations. Moreover, long‐term stability tests confirmed the robustness of the MoP pillars, which maintained a high FE (>90%) and a stable current density over 80 h of continuous operation, underscoring their potential for continuous H_2_O_2_ electrosynthesis. Overall, this study provides valuable insights into the structure‒performance relationships of MoP catalysts, emphasizing the critical role of crystallographic facet control in optimizing catalytic performance. Our findings not only position MoP as an efficient and selective electrocatalyst for H_2_O_2_ production but also open up new avenues for the rational design of advanced TMP‐based materials for applications in diverse contexts, including catalysis, and with further modifications, in batteries, fuel cells, and other functional devices.

## 4 Experimental Section

### CVD Growth of MoP Single Crystals on Liquid Ga

A commercial Ga pellet (Alfa Aesar, >99.9% purity) was divided into small Ga droplets. Mo foil (Alfa Aesar, 99.95 wt.% purity) was rinsed with ethanol. A Ga droplet was placed on the Mo foil (1 cm × 1 cm) to form the Ga–Mo substrate, which was then heated in a quartz tube (diameter: 1 in) to 700 °C–900 °C at a rate of 30 °C min^−1^. Red phosphorus powder (Alfa Aesar, >99.9% purity) was then introduced into the tube furnace, and the temperature was maintained at 500 °C. MoP growth proceeded over 30 min under Ar (250 sccm) and H_2_ (50 sccm) flow. Finally, the substrates with grown MoP samples were rapidly cooled to room temperature.

### Characterizations

The obtained samples were examined using SEM (ZEISS Merlin Compact SEM) and XPS (Thermo Scientific, ESCALAB 250Xi, monochromatic Al Kα radiation). Elemental mapping was performed using EDS. Raman spectra were acquired at room temperature using a Raman spectrometer (XperRAM S, Nanobase) with a 532 nm laser, a beam diameter of 1 µm, and ×40 magnification. Raman spectra were recorded using gratings with 1800 grooves mm^−1^, and the Raman peak of Si at 521 cm^−1^ was used as the reference for wavenumber calibration. XRD measurements were performed using an X‐ray diffractometer (D8 FOCUS; Bruker). Absorbance spectra were recorded at room temperature with a JASCO V‐770 spectrophotometer using an 808 nm laser. The EBSD image was acquired using an environmental scanning electron microscope (Quattro S, Thermo Fisher) equipped with EDAX Velocity EBSD camera.

### Electrochemical Characterization

Measurements of electrochemical H_2_O_2_ production were conducted using a conventional three‐electrode cell with an RRDE setup and an Autolab potentiostat (PGSTAT302N). A platinum sheet (Autolab) served as the CE, while an Ag/AgCl electrode functioned as the reference electrode. The RRDE WE consisted of a glassy carbon disk (0.196 cm^2^ area) and a platinum ring. Before measurement, the WE was mechanically polished with alumina powder and rinsed with deionized (DI) water. A catalyst slurry was prepared by mixing the synthesized catalyst (3 mg) with 0.475 µL of isopropanol, 1.425 µL of DI water, and 50 µL of a 5% Nafion solution (5 wt%), followed by ultrasonication for 25 min to create a homogeneous ink. The resulting catalyst ink was drop‐casted onto the glassy carbon electrode at a loading of 20 µg cm^−2^. A thin catalyst layer was formed to ensure that the formed H_2_O_2_ was detectable at the ring electrode. Once the solvent was evaporated, the WE was deemed ready for experimentation. Before catalytic performance testing, precycling was performed using cyclic voltammetry (CV). Here, the catalyst was cycled ten times in an Ar‐saturated 0.1 M KOH solution with a scan rate of 0.1 V s^−1^ and a scan range of 0.05‒1.0 V. The platinum ring surface was cleaned via CV over 20 cycles at a scan rate of 0.5 V s^−1^ and a scan range of 0.05‒1.0 V. The H_2_O_2_ production rate and selectivity were measured in an O_2_‐saturated electrolyte at a scan rate of 0.01 V s^−1^ and a rotation rate of 1600 rpm for the disk electrode. Notably, the platinum ring electrode was maintained at 1.2 V (versus RHE), ensuring the exclusive oxidation of H_2_O_2_ generated on the disk electrode. The ORR current was corrected by subtracting the current obtained under Ar‐saturated conditions from the current measured in the O_2_‐saturated electrolyte. Meanwhile, the ring current was corrected using the RRDE collection efficiency to ensure accurate quantification of H_2_O_2_ generation. The collection efficiency of the electrode was determined using a potassium hexacyanoferrate (III) solution (N = 0.278, Figure S11, Supporting Information).

The following equations were used to calculate the collection efficiency, selectivity, 2e^−^ mass activity, TOF, and kinetic current density.

(1) $$\mathrm{Selectivity}\left(\%\right)=\frac{200\times \left(I_{r}/N\right)}{\left(I_{d}+I_{r}/N\right)}\;$$

(2) $$2e^{-}\;\mathrm{mass}\;\mathrm{activity}\;=\frac{\mathrm{Ring}\;\mathrm{current}\;\mathrm{density}\;\left[A/cm^{2}\right]}{\mathrm{Catalyst}\;\mathrm{loading}\;\mathrm{density}\;\left[g/cm^{2}\right]}$$

(3) $$\mathrm{Turnover}\;\mathrm{frequency}\;\left[s^{-1}\right]=\frac{\mathrm{number}\;\mathrm{of}\;\mathrm{oxygen}\;\mathrm{molecules}\;\mathrm{turnover}}{\mathrm{number}\;\mathrm{of}\;\mathrm{active}\;\mathrm{sites}}\;$$

(4) $$\begin{array}{c} \mathrm{Number}\;\mathrm{of}\;\mathrm{oxygen}\;\mathrm{molecules}\;\mathrm{turned}\;\mathrm{over}\;=\;j\left[\frac{\mathrm{mA}}{cm^{2}}\right]\times \frac{1\left[\frac{C}{s}\right]}{1000\left[\mathrm{mA}\right]}\\ \times \frac{1\left[\mathrm{mol}\;e^{-}\right]}{96485\left[C\right]}\times \frac{1\left[\mathrm{mol}\;O_{2}\right]}{2\left[\mathrm{mol}\;e^{-}\right]}\times \left(6.02\times 10^{23}\left[\frac{\mathrm{atom}\;O_{2}}{\mathrm{Mol}\;O_{2}}\right]\right) \end{array}$$

(5) $$\begin{array}{c} \mathrm{Number}\;\mathrm{of}\;\mathrm{active}\;\mathrm{sites}=\;L\;\left[\frac{\mathrm{mg}}{{\mathrm{cm}}^{2}}\right]\times R\;\left[\mathrm{wt}\%\right]\times \frac{1\left[\mathrm{mmol}\right]}{W\;\left[\mathrm{mg}\right]}\\ \times \left(6.02\times 10^{20}\left[\frac{\mathrm{atom}}{\mathrm{mmol}}\right]\right) \end{array}$$

In the above equations, *I_r_* denotes the ring current, *I_d_* represents the disk current, and *N* denotes the current collection efficiency (0.278). The parameter *j* signifies the current density for H_2_O_2_ production, measured from the ring electrode using the collection efficiency of the RRDE setup at a given overpotential. L (0.02 mg cm^−2^)  denotes the amount of catalyst loaded on the electrode, R (0.244 wt%) represents the weight fraction, and W(30.97 mg) signifies the atomic weight of the active site element.

(6) $$j_{k}=\frac{j\times j_{d}}{j_{d}-j}\;,$$

(7) $$\frac{1}{j}=\frac{1}{j_{k}}+\frac{1}{0.62nFC_{0}\left(D_{0}\right)2/3v^{-1/6}w^{1/2}},$$

where *j_k_* denotes the kinetic current density, *j* represents the measured current density, *w* denotes the angular velocity, *n* represents the electron transfer number, *F* denotes the Faraday constant (96 485 C mol^−1^), *C*_0_ represents the saturated concentration of O_2_ in the electrolyte at room temperature (1.1 × 10^−6^ mol cm^−3^), *D*_0_ denotes the diffusion coefficient of O_2_ in the electrolyte (1.9 × 10^−5^ cm^2^ s^−1^), and *v* represents the kinematic viscosity of the electrolyte at 25 °C (1 × 10^−2^ cm^2^ s^−1^).

The ECSA was determined using the double‐layer capacitance method. In the non‐Faradaic region, constant potential cyclic voltammetry (CV) scans were performed at scan rates of 60, 80, 100, 120, 140, and 160 mV s^−1^ in 0.1 M Ar‐saturated KOH to exclude the effect of trace oxygen in the electrolyte. By plotting Δ*j* as a function of scan rate, the slope – corresponding to the double‐layer capacitance (*C_dl_*) – was obtained, which is linearly related to the ECSA. ECSA was then calculated using the following equations:

(8) $$\mathrm{ECSA}\;=\frac{C_{dl}}{C_{s}}\times A_{GCE}$$

where *C_s_* is a specific capacitance for the electrode surface and *A_GCE_* is an electrode surface area.^[^ ^49^ ^]^ The typical *C_s_* value of 40 µF cm^−2^ for ECSA calculation was adopted.^[^ ^50^, ^51^ ^]^

### Accumulated H_2_O_2_ Measurement in an H‐Cell System

Measurements of electrocatalytic H_2_O_2_ production were conducted using a Teflon‐treated carbon fiber paper loaded with the catalysts in a two‐compartment H‐cell system with a Nafion 117 membrane as the separator. The setup included an Ag/AgCl reference electrode, a graphite rod as the CE, and MoP‐pillar‐modified carbon fiber paper (0.5 × 0.5 cm^2^) as the WE, with a mass loading rate of 0.1 mg cm^−2^. Both the cathode (50 mL) and anode compartments were filled with the same electrolyte.

The concentration of produced H_2_O_2_ was determined using a colorimetric method. For calibration, H_2_TiO_4_ standards were prepared by treating H_2_O_2_ solutions (concentration range: 0.1‒4.0 mM) with 3.8 mL of 0.1 M acidified titanium sulfate. These standards were examined using an ultraviolet‒visible spectrophotometer at a wavelength of 407 nm. The standard concentrations and corresponding absorbances at 407 nm are illustrated in Figure S13 (Supporting Information). A linear calibration curve was created for H_2_O_2_ concentrations ranging from 0.1‒4.0 mM. Notably, this calibration is applicable to acidified titanium sulfate solutions within this concentration range. To determine the concentration of H_2_O_2_ in the electrolyte, 0.2 mL of the collected electrolyte was reacted with 3.8 mL of a 0.1 M acidified titanium sulfate solution, and the results were compared to the linear calibration curve. The total charge flux through the electrode was integrated over time, demonstrating high FE for the instantaneous production of H_2_O_2_ and high efficiency for its continuous production using chronoamperometry. This high efficiency indicates the potential of the system for long‐term H_2_O_2_ production.

H_2_O_2_ productivity was calculated using the following equations:

(9) $$\mathrm{FE}\;=2\times C_{H_{2}O_{2}}\times F\times \frac{V}{Q}\times 100\%,$$

(10) $$H_{2}O_{2}\;\mathrm{productivity}\;\;\left[\mathrm{mmol}\;h^{-1}g^{-1}\right]=C_{H_{2}O_{2}}\times \;V\times \frac{10^{3}}{A\times m\times t},$$

where $C_{H_{2}O_{2}}$ denotes the concentration of H_2_O_2_ (0.00 035797 mol L^−1^), *F* represents the Faraday constant (96 485.3 C mol^−1^), *V* denotes the electrolyte volume (0.5 L), *Q* represents the accumulated charge (4.70208 C), *A* signifies the electrode area (0.5 cm^2^), *m* represents the catalyst mass loading (0.1 mg cm^−2^), and *t* indicates the reaction time (1 h).

### Computational Details

All DFT calculations were performed using the Vienna Ab initio Simulation Package.^[^ ^52^, ^53^, ^54^ ^]^ The generalized‐gradient approximation^[^ ^55^ ^]^ with the Perdew‒Burke‒Ernzerhof^[^ ^56^ ^]^ functional was employed to describe the exchange‐correlation energy. A projector‐augmented wave^[^ ^57^ ^]^ pseudopotential was used to represent electron‒ion interactions. The Mo 4s^2^4p^6^4d^5^5s^1^, P 3s^2^3p^3^, O 2s^2^2p^4^, and H 1s^1^ electrons were explicitly treated as valence states. A plan‐wave basis set was applied, and the kinetic energy cutoff was set at 500 eV. The convergence criteria for total electronic energy and structural optimization forces were set to 10^−6^ eV and 0.02 eV Å^−1^, respectively. Brillouin zones were sampled using a 12 × 12 × 9 Monkhorst‒Pack k‐point grid for bulk models and a 3 × 3 × 1 grid for slab models.^[^ ^58^ ^]^

According to the XRD measurements, the (0001) surface dominates the nanoplate morphology, while the $\left(10\bar{1}0\right)$ surface dominates the pillar structure. The XRD profiles also include a prominent peak corresponding to the $\left(10\bar{1}1\right)$ surface. To determine the surface structures, the surface free energies of the (0001), $\left(10\bar{1}0\right)$, and $\left(10\bar{1}1\right)$ surfaces were calculated, as shown in Figures S1 and S17 (Supporting Information).

For accurate surface free energy calculations, one side of the slab was fixed, while the other side was allowed to relax. This approach, based on the method proposed in a previous study,^[^ ^32^ ^]^ differs from conventional methods and is described by the following equations:

(11) $$\gamma =\frac{E_{cle}+E_{rel}}{A}$$

(12) $$E_{cle}=\frac{E_{unrelax}-N_{Mo}\times E_{bulk}-\left(N_{Mo}-N_{P}\right)\times \mu_{P}}{2}$$

If the surface is symmetric (e.g., (0001) and $\left(10\bar{1}1\right)$), we have

(13) $$E_{rel}=\frac{E_{relax}-E_{unrelax}}{2}$$

If the surface is asymmetric (e.g., $\left(10\bar{1}0)\right)$, we have

(14) $$E_{rel}=E_{relax}-E_{unrelax}$$

In the above equations, *E_unrelax_* and *E_relax_* represent the total energies of the unrelaxed and partially relaxed (upper half) MoP surfaces, respectively. *A* denotes the area of the slab model, *N* is the number of atoms, and µ represents the chemical potential. For the slab models, the bottom three layers were fixed in the (0001) and $\left(10\bar{1}0\right)$ models, each with nine layers, while the bottom two layers were fixed in the $\left(10\bar{1}1\right)$ model, consisting of five layers. A vacuum region of 15 Å was added to the slab model system to minimize interactions between adjacent surfaces caused by period boundary conditions.

The diffusion energy barrier of a Mo adatom on the (0001) surface of MoP was calculated using the climbing image nudged elastic band (CI‐NEB) method with a spring constant between images of ‐5.0 eV Å^−2^ and a force convergence criterion of 0.05 eV Å^−1^.^[^ ^59^, ^60^ ^]^ A MoP slab model with a stepped (0001) surface was constructed to simulate lateral diffusion across a terrace and step edge. The minimum energy pathway for Mo adatom migration was determined by interpolating a series of intermediate images between the initial and final states.

The 2e^−^ pathway of the ORR, which leads to H_2_O_2_ production, involves the following reactions.

(15) $$*\;+O_{2}\left(g\right)+H^{+}+e^{\_}\to \;*\mathrm{OOH}$$

(16) $$*\mathrm{OOH}\;+H^{+}+e^{\_}\to H_{2}O_{2}\left(g\right)$$

Conversely, the 4e^−^pathway of the ORR in an acidic solution proceeds as follows.

(17) $$*\;+O_{2}\left(g\right)+H^{+}+e^{-}\to \;*\mathrm{OOH}$$

(18) $$*\mathrm{OOH}\;+H^{+}+e^{-}\to \;*O\;+H_{2}O$$

(19) $$*O\;+H^{+}+e^{-}\to \;*\mathrm{OH}$$

(20) $$*\mathrm{OH}\;+H^{+}+e^{-}\to H_{2}O\;\left(l\right)$$

The adsorption energy was calculated using the following equation:

(21) $$E_{\mathrm{ads}}=E_{\mathrm{surface}+\mathrm{adsorbate}}^{\mathrm{tot}}\;-E_{\mathrm{surface}}^{\mathrm{tot}}-\mu_{\mathrm{adsorbate}}$$

where $E_{\mathrm{surface}+\mathrm{adsorbate}}^{\mathrm{tot}}$, $E_{\mathrm{surface}}^{\mathrm{tot}}$, and µ_adsorbate_ represent the total energy of the slab model with the adsorbate attached to the surface, the total energy of the surface slab model, and the chemical potential of the adsorbed material, respectively.

Subsequently, the Gibbs free energy (Δ*G*_ads_) was calculated using the following equation, based on the calculated adsorption energy:

(22) $$\Delta \;G_{ads}=E_{ads}+\Delta ZPE-T\Delta S$$

where *E*_ads_ represents the adsorption energy, calculated using Equation (21), Δ*ZPE* denotes the zero‐point energy difference between the surface and adsorbed molecule, *T* represents the temperature, and Δ*S* indicates the entropy change. These values for the adsorbed molecules were obtained through DFT calculations at room temperature.

### Statistical Analysis

Any pre‐processing of data is described in the relevant methods section above. Data is presented as mean ± standard deviation throughout. Size analysis was performed using ImageJ software. No additional statistical tests were performed in this study.

## Conflict of Interest

The authors declare no conflict of interest.

## Author Contributions

S.H.K., J.‐H.K., and B.P. contributed equally to this work. S.H.K., J.W.J., and H.J.H. develop synthesis. B.P. performed the computational calculations and data analysis. J.‐H.K. and J.‐G.L. performed electrochemical characterizations. B.P. performed computational calculations and data analysis. B.‐H.K. conceived computational strategy and supervised computational studies and data analysis. S.Y. measure the XPS. H.J. performed EBSD analysis. H.J.H. and M.‐J.C. conceived the concept. H.J.H. led the discussions. S.H.K., S.K., B.‐H.K., M.‐J.C., and H.J.H. contributed to the discussion of the content and writing of the manuscript. All authors have read and agreed to the published version of the manuscript.

## Supporting information



Supporting Information

## Data Availability

The data that support the findings of this study are available from the corresponding author upon reasonable request.
