# Supplementary material for: Facet‐Controlled Growth of Molybdenum Phosphide Single Crystals for Efficient Hydrogen Peroxide Synthesis
Source: Adv Mater. 2025 May 28;37(34):2500250. doi: 10.1002/adma.202500250 (PMC12392870; doi:10.1002/adma.202500250)
Supplement: Supplementary file 1 — Supporting Information [file ADMA-37-2500250-s001.docx]

Supporting Information

Facet-Controlled Growth of Molybdenum Phosphide Single Crystals for Efficient Hydrogen Peroxide Synthesis

Seo Hyun Kim, Jeong-Hyun Kim, Bogeun Park, Hanhwi Jang, Jeong-Gyu Lee, Soonmin Yim, Jae Won Jeong, Seyoung Koo, Yeon Sik Jung, Byung-Hyun Kim*, Min-Jae Choi* and Hyeuk Jin Han*

**Note S1. Synthesis of Polycrystalline MoP Powder**

To synthesize MoP powder, MoO₃ powder (Sigma–Aldrich, 99.95%) was converted to MoP by phosphine gas. The MoO₃ powder was placed inside a tube furnace. Upstream, approximately 2 grams of NaH₂PO₂·H₂O (Sigma-Aldrich, purity ≥99%) were positioned about 15 to 17 cm away from the furnace's center. After purging the system with argon, it was evacuated to a pressure of 200 mTorr. Then, hydrogen gas was introduced at a flow rate of 20 sccm, increasing the pressure inside the furnace to atmospheric levels. The furnace was heated to 800 °C over 30 minutes, maintained at this temperature for one hour, and then allowed to cool naturally back to room temperature.

**
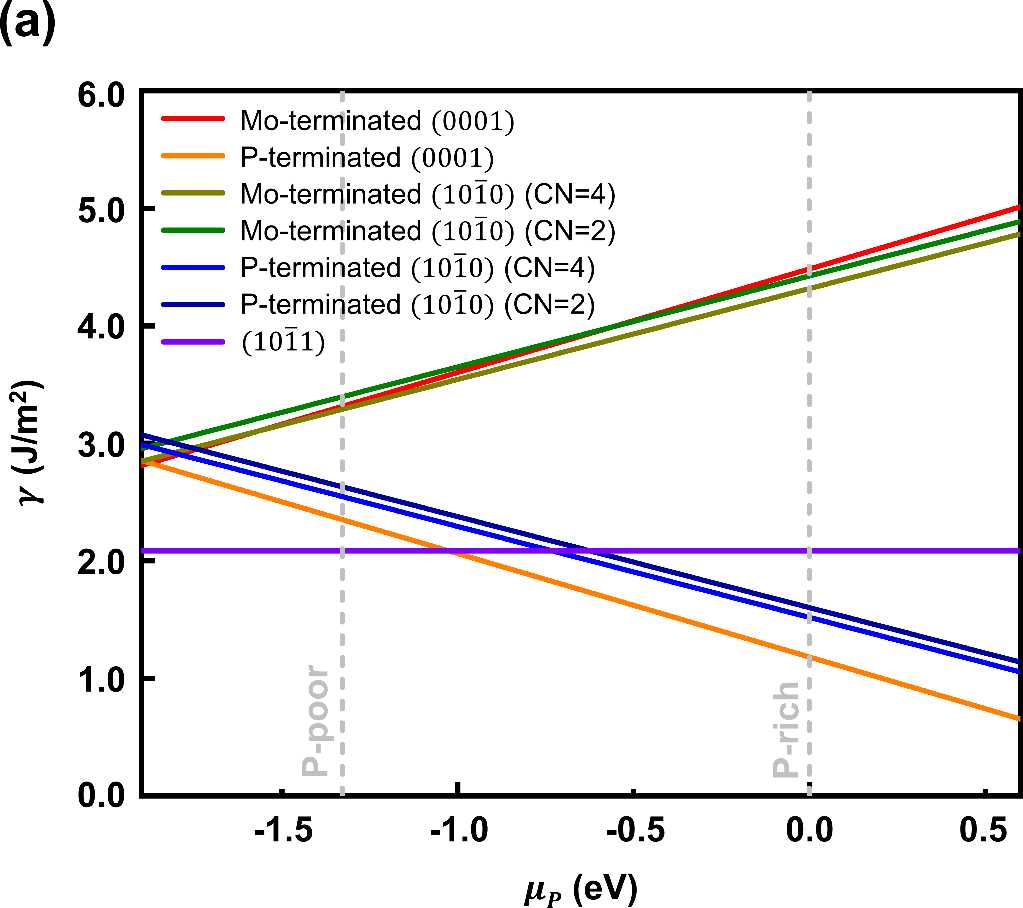
**

**Figure S1.** Surface free energies of MoP as a function of the chemical potential of P, with different orientations, terminations and coordination numbers.

**
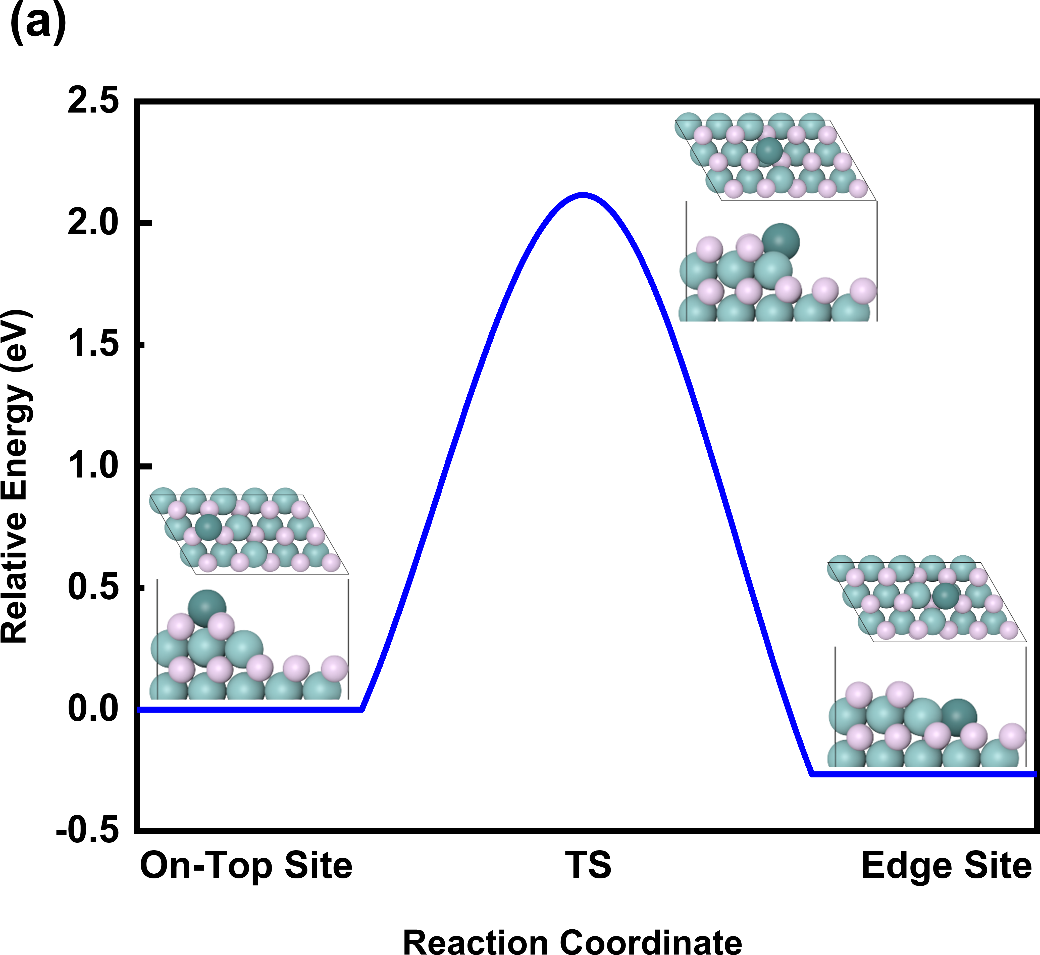
**

**Figure S2.** DFT-calculated diffusion energy barrier of a Mo adatom on the $(0001)$ surface of MoP. The energy profile represents the migration of a Mo adatom from the terrace to a lower step edge, illustrating the energy barrier for lateral growth on the $(0001)$ surface.

**
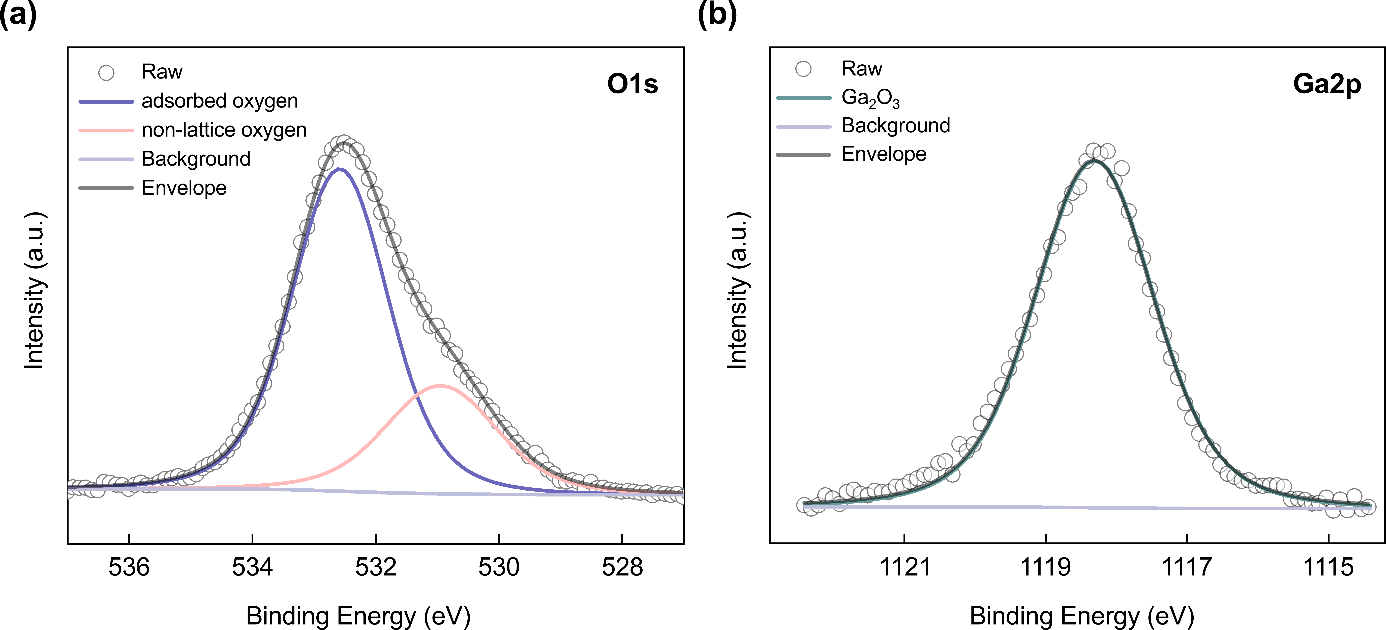
**

**Figure S3.** X-ray photoelectron spectroscopy spectra of as-synthesized MoP single crystals showing the (a) O 1s (b) Ga 2p peaks.

**
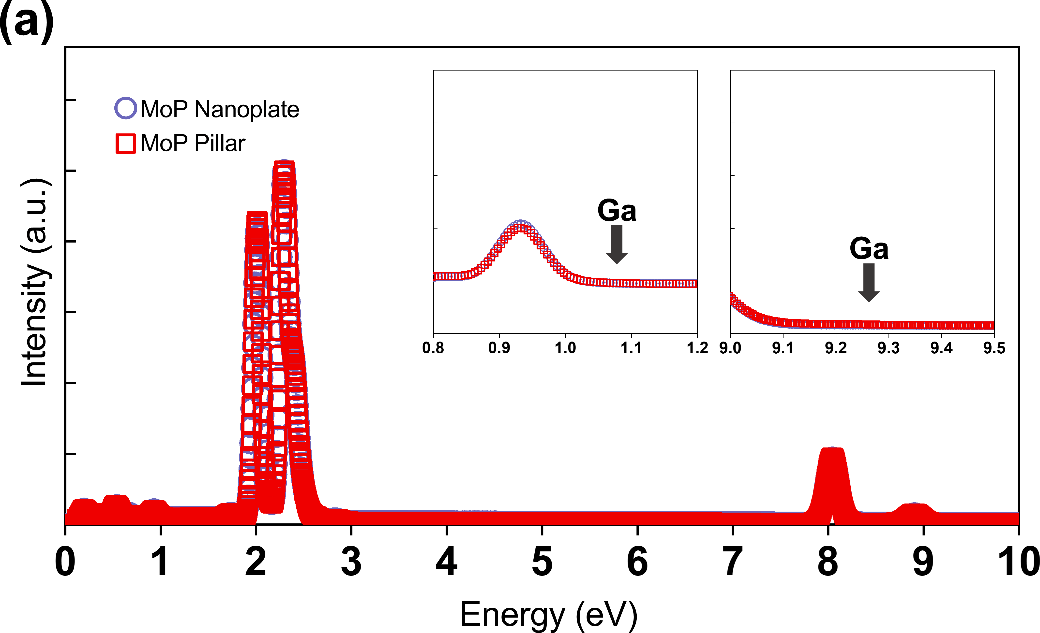
**

**Figure S4.** Energy Dispersive X-ray Spectroscopy analysis of MoP crystals using Transmission Electron Microscopy. The results show no detectable gallium (Ga) within individual crystals, with a detection limit of approximately 0.5 at%. This confirms that the MoP crystals are intrinsically free of Ga impurities.


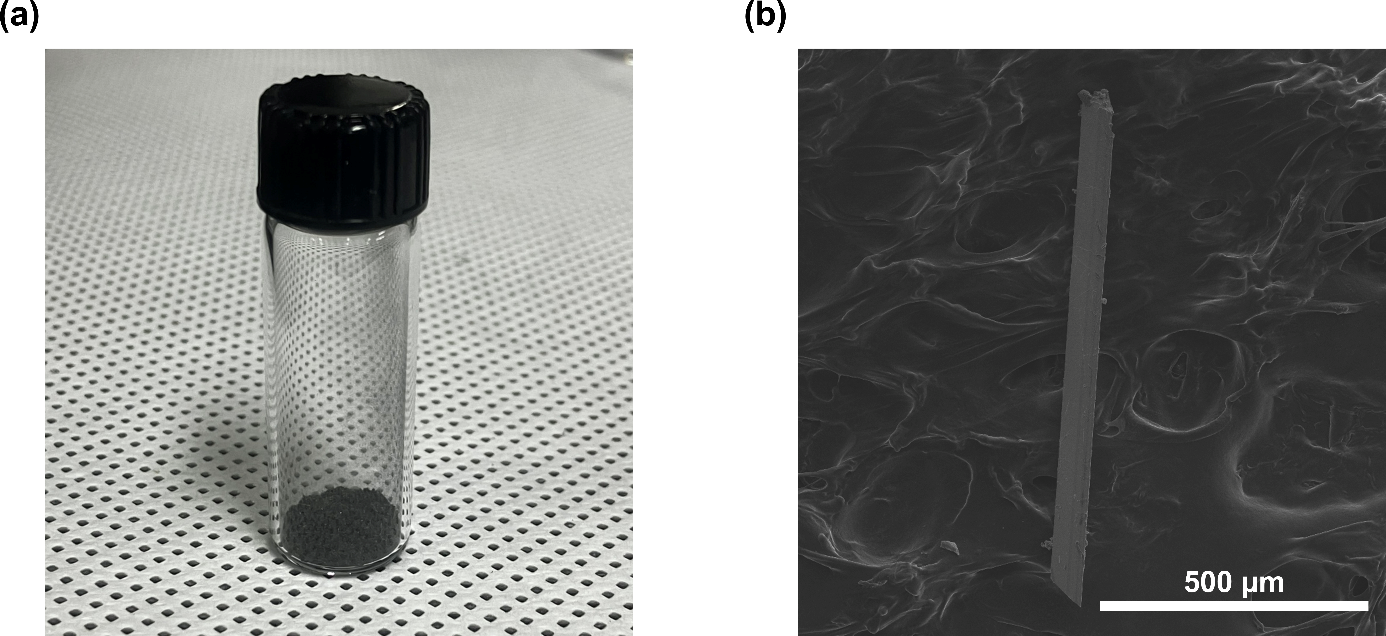


**Figure S5.** Comparison of crystal production efficiency between our synthesis method and the Chemical Vapor Transport method. (a) amount of crystals synthesized using our method, which achieves a remarkable production efficiency (~ 60 mg/hr). (b) MoP_2_ crystals synthesized using the CVT method.

**
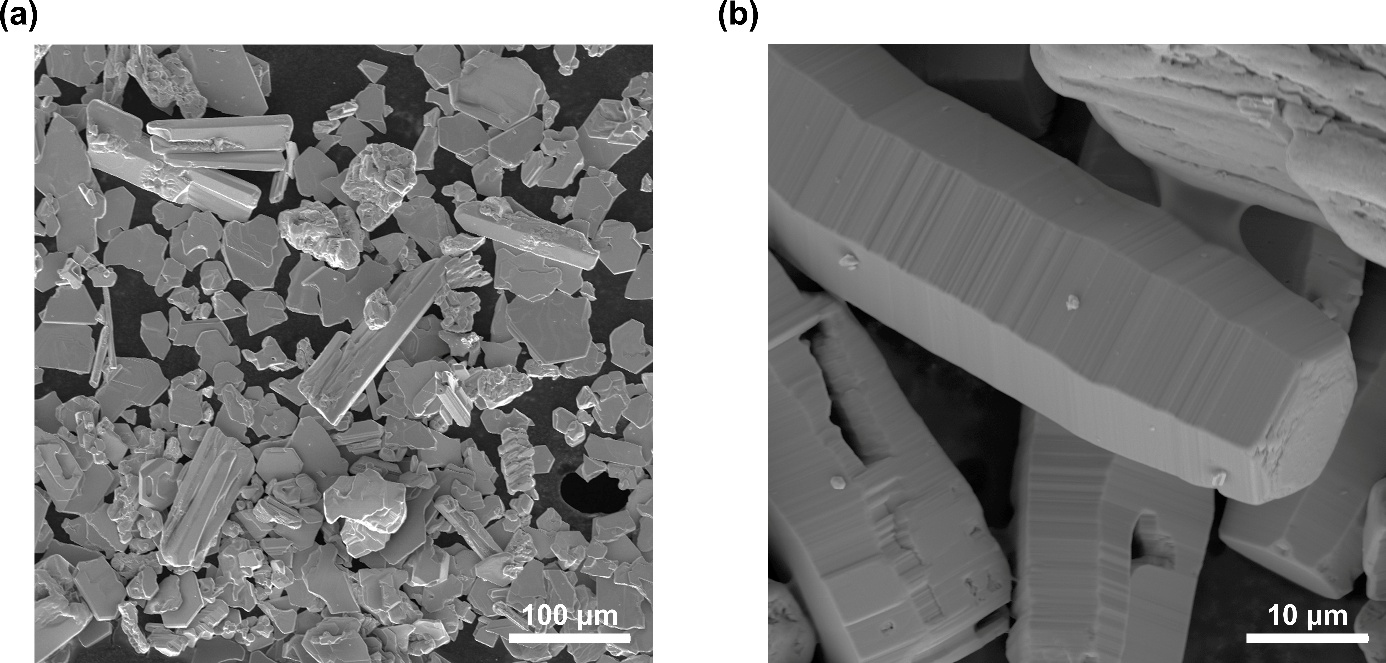
**

**Figure S6.** SEM images of (a) the MoP nanoplates and (b) the MoP pillars, collected from the electrode surface. The MoP nanoplates exhibit a hexagonal morphology, while the MoP pillars display an elongated structure with a $(10\bar{1}0)$ facet orientation. These structural features are consisten with their prisinte morphology shown in Figure 2a and Figure 2d, respectively.


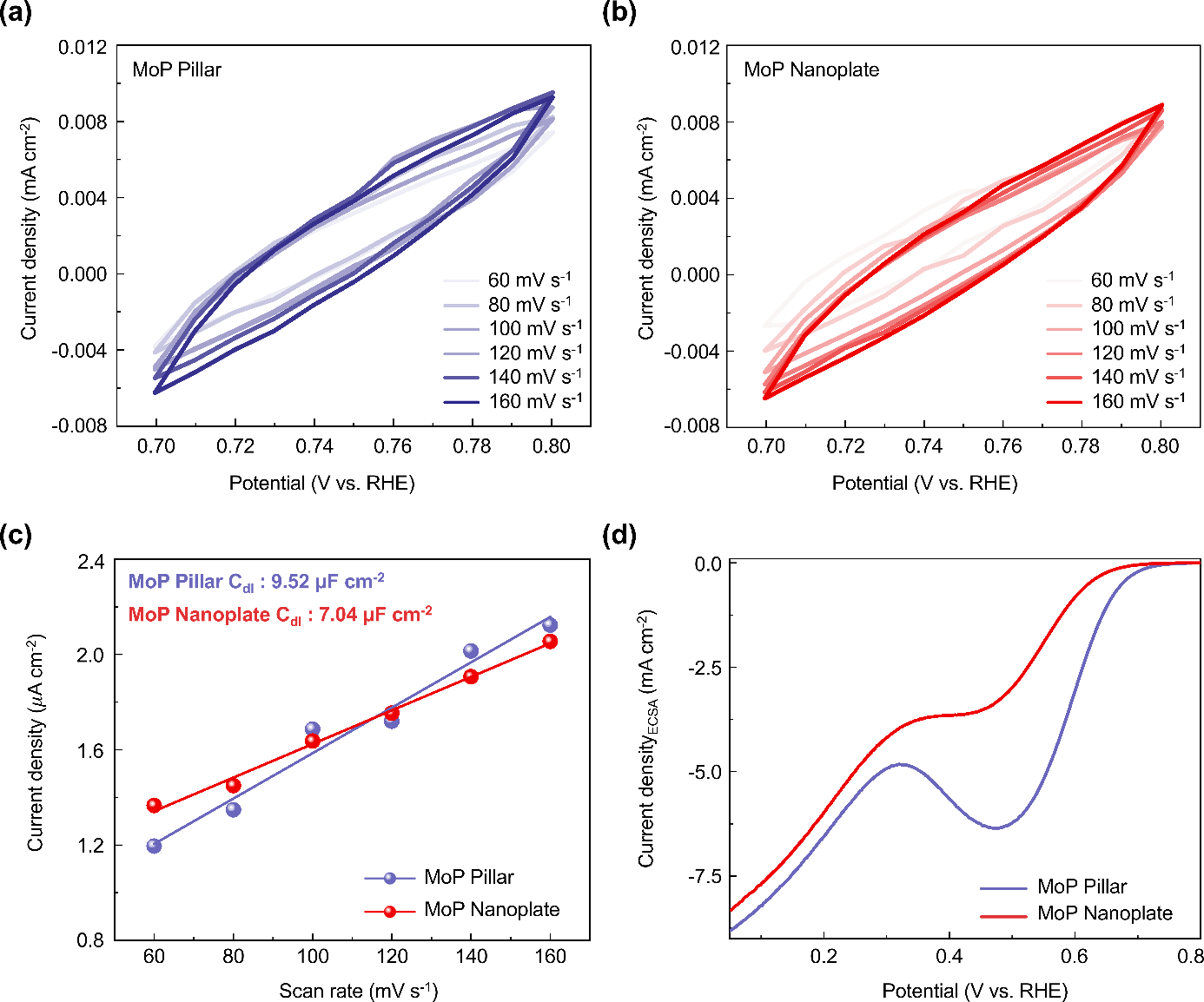


**Figure S7.** (a, b) CV curves of the MoP pillar (a) and the nanoplate (b) at the scan rates ranging from 60 mV s^-1^ to 160 mV s^-1^ within a potential window of 0.7 V-0.8 V (vs. RHE). (c) The double-layer capacitance (C_dl_) determined from the CV curves. (d) The LSV curves normalized by ECSA.

**
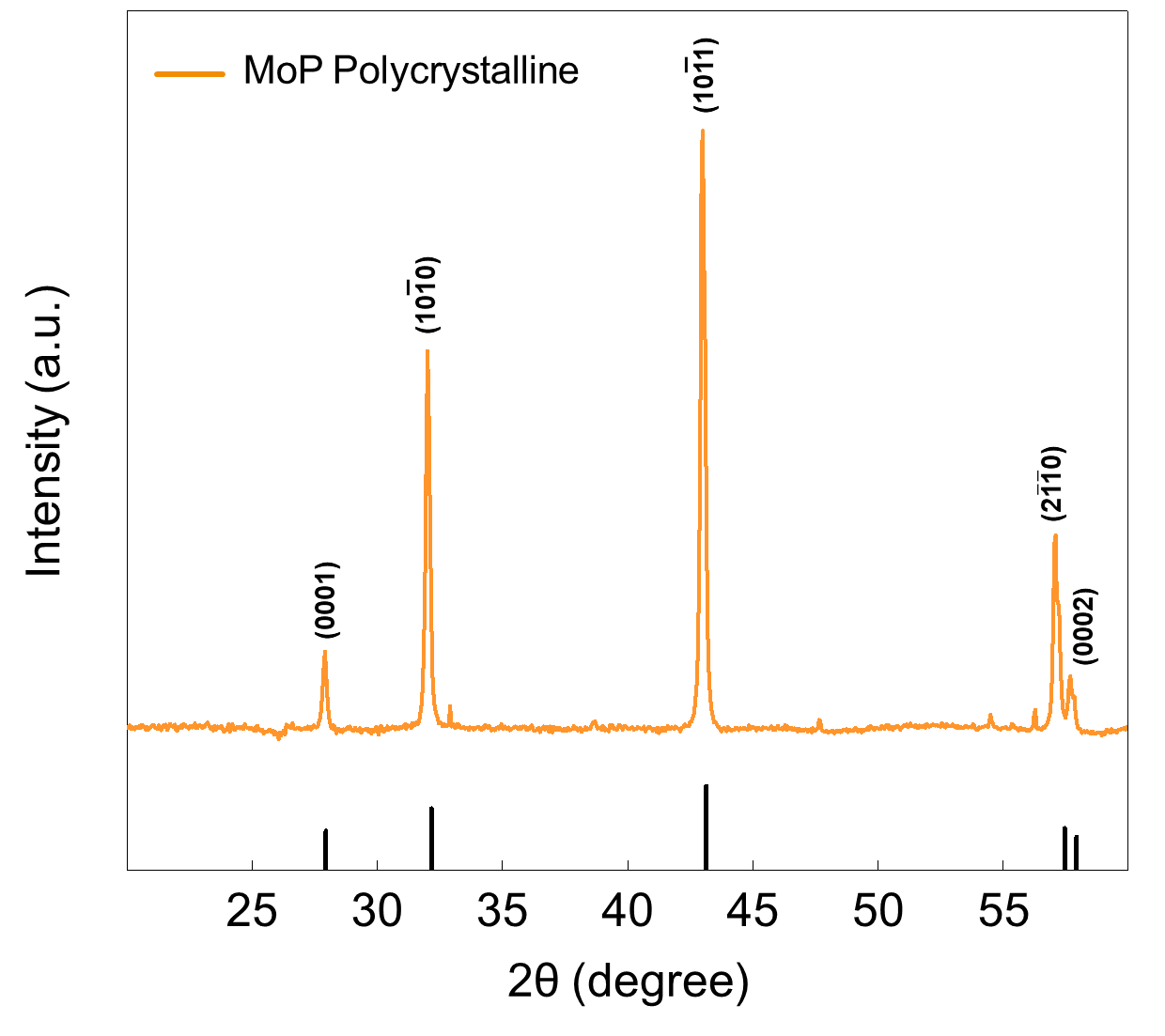
**

**Figure S8.** X-ray diffraction (XRD) patterns of polycrystalline MoP.


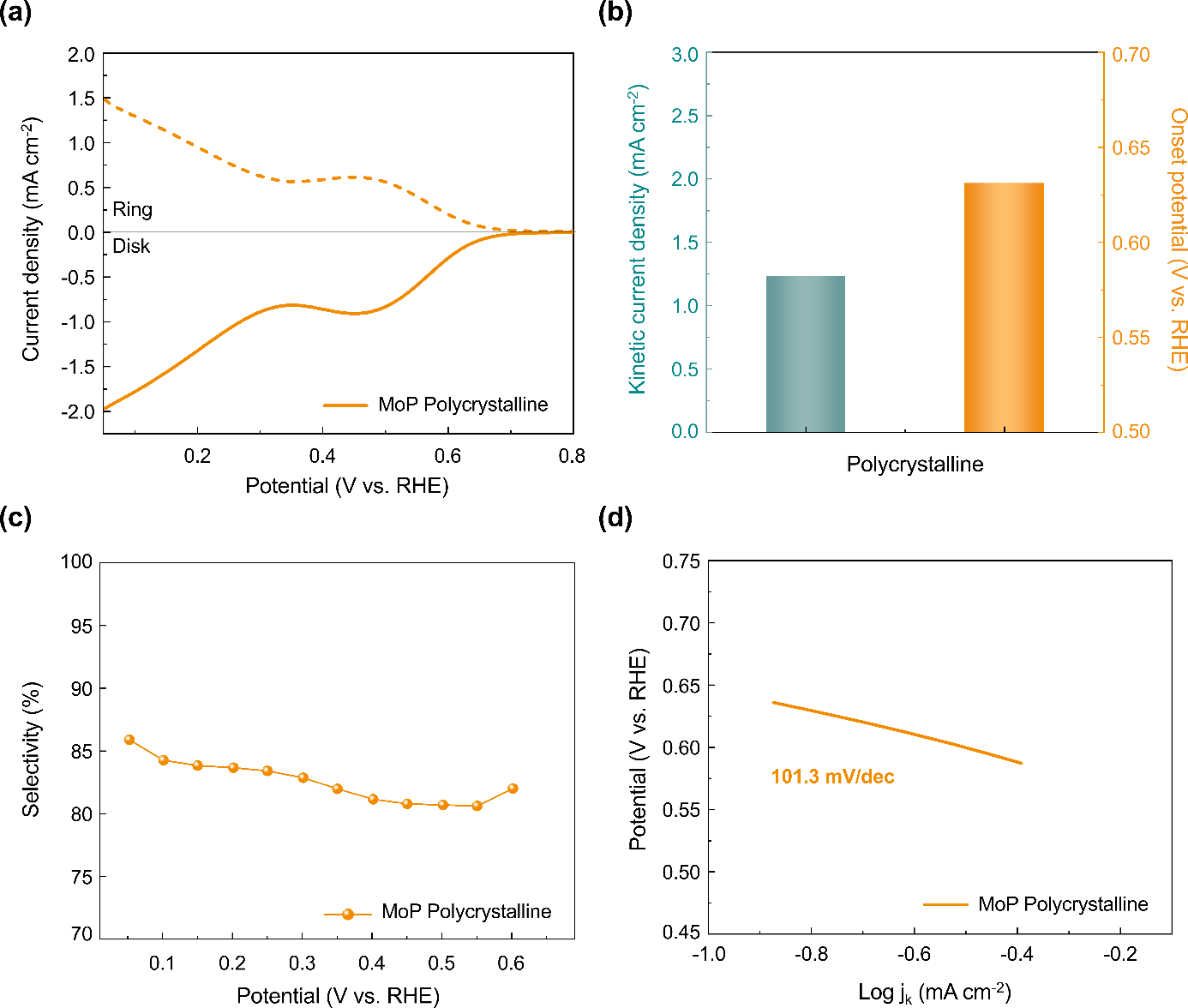


**Figure S9. Electrochemical ORR performances of polycrystalline catalysts for H_2_O_2_ production.** (a) ORR performance and the simultaneous H_2_O_2_ detection current densities at the ring electrode for polycrystalline catalysts in O_2_-saturated 0.1 M KOH at a sweep rate of 10 mV/s. (b) kinetic current density (at 0.45 V vs. RHE) and onset potential (at disk current density 0.1 mA/cm^2^) (c) Calculated H_2_O_2_ selectivity. (d) Mass-transfer-corrected Tafel plots of kinetic current densities for H_2_O_2_ production.


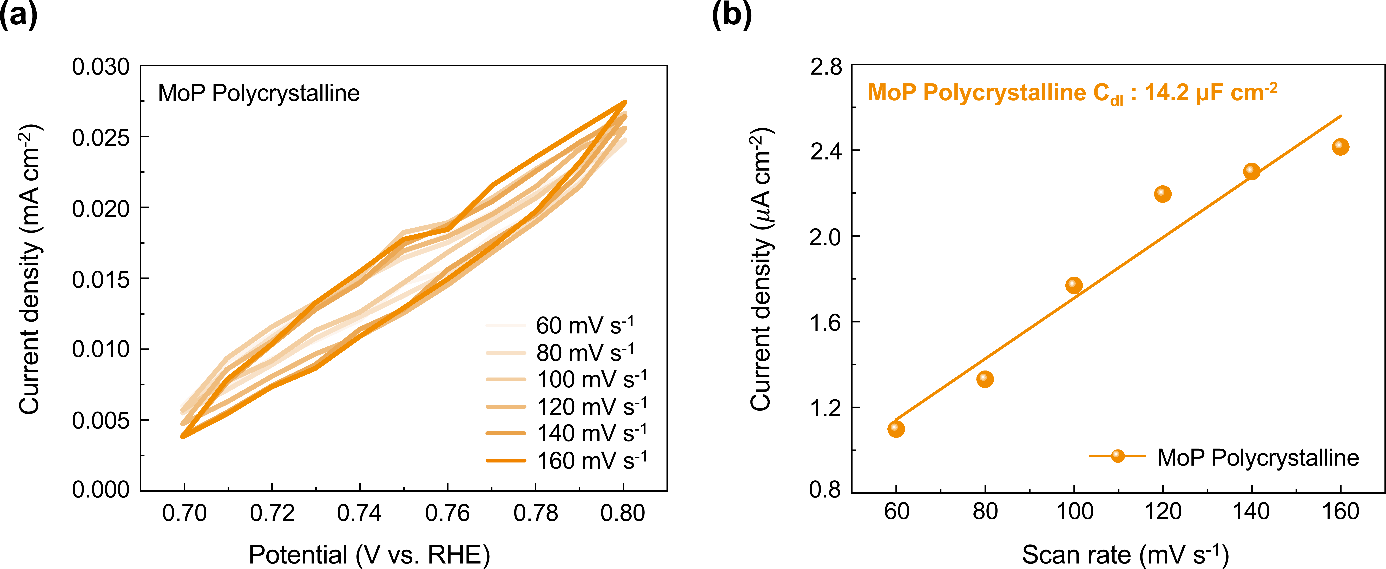


**Figure S10.** CV curves at different scan rates and the calculated ECSAs. (a) The CV curves were measured at the scan rates of 60 mV s^-1^ to 160 mV s^-1^ in the potential window of 0.7 V-0.8 V (vs. RHE) for the polycrystalline MoP. (b) According to the double-layer capacitance (C_dl_) method based on the CV curves, the ECSAs can be calculated.

**
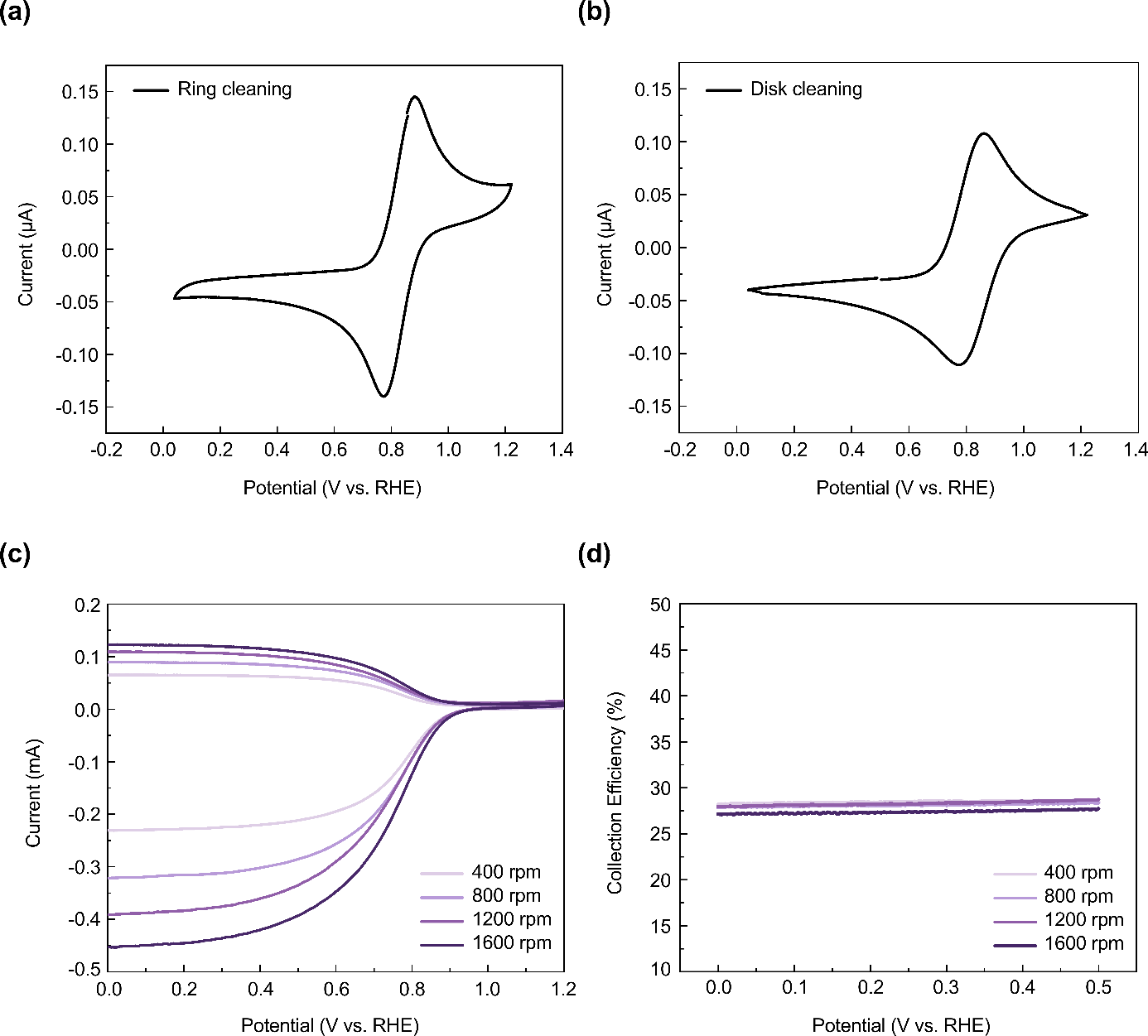
**

**Figure S11.** Calibration of the collection efficiency of the bare RRDE in Ar-saturated 0.05 M Na_2_SO_4_ dissolved with 4 mM of K_3_[Fe(CN)_6_]. Clean the bare GC disk and Pt ring to obtain accurate measurements; (a) Cyclic voltammograms of the Pt ring of RRDE at 100 mV/s and 0 rpm, (b) CV voltammetry of the bare GC disk of RRDE at 100 mV/s and 0 rpm, (c) RRDE voltammograms recorded at different rotation rates by performing LSV on the disk from 1.23 V to 0 V vs. RHE at 50 mV/s while holding the ring at 1.20 V vs. RHE. (d) the corresponding collection efficiency of RRDE voltammograms as a function of potential. All potential in this figure is presented without i_R_-correction.

**
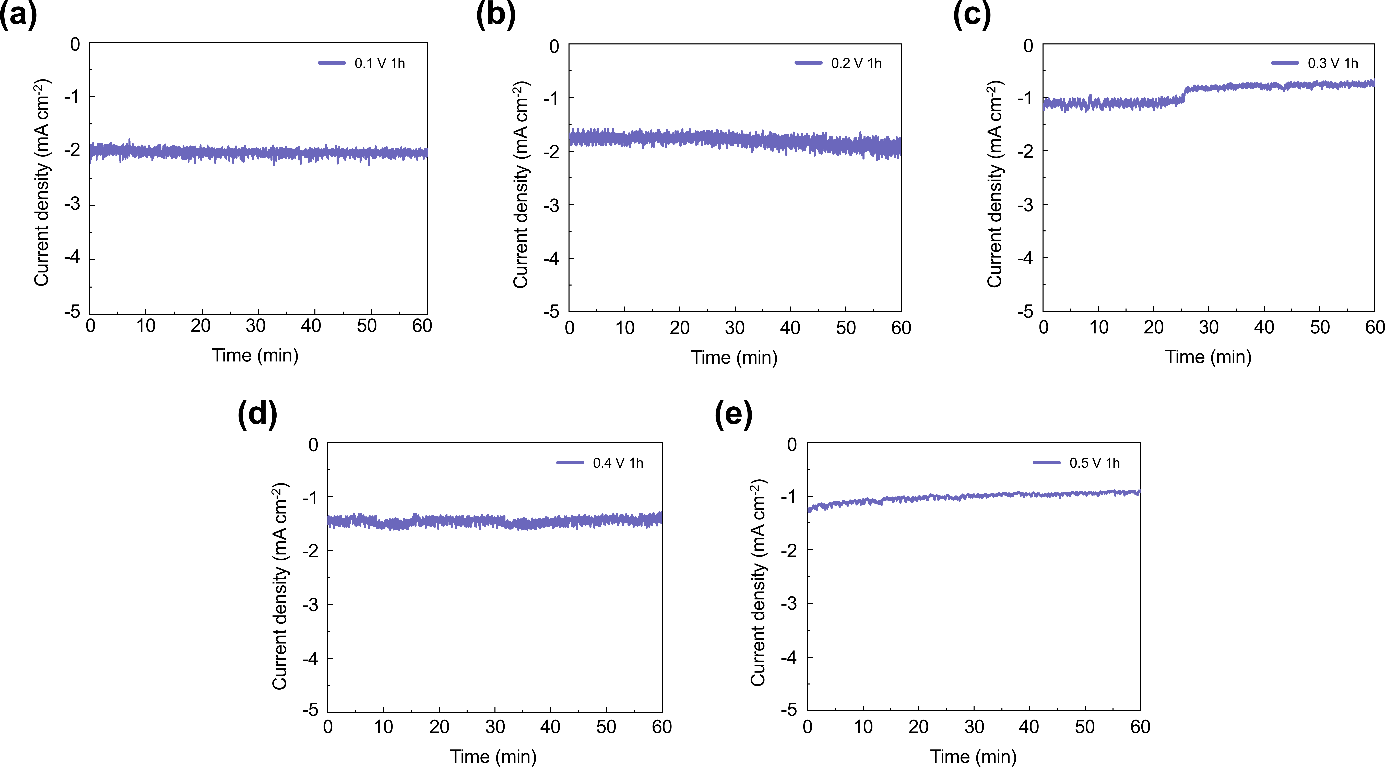
**

**Figure S12.** Current density of MoP pillar measured at different voltages (0.1 V (a), 0.2 V (b), 0.3 V (c), 0.4 V (d), and 0.5 V (e)) for 1 hour.

**
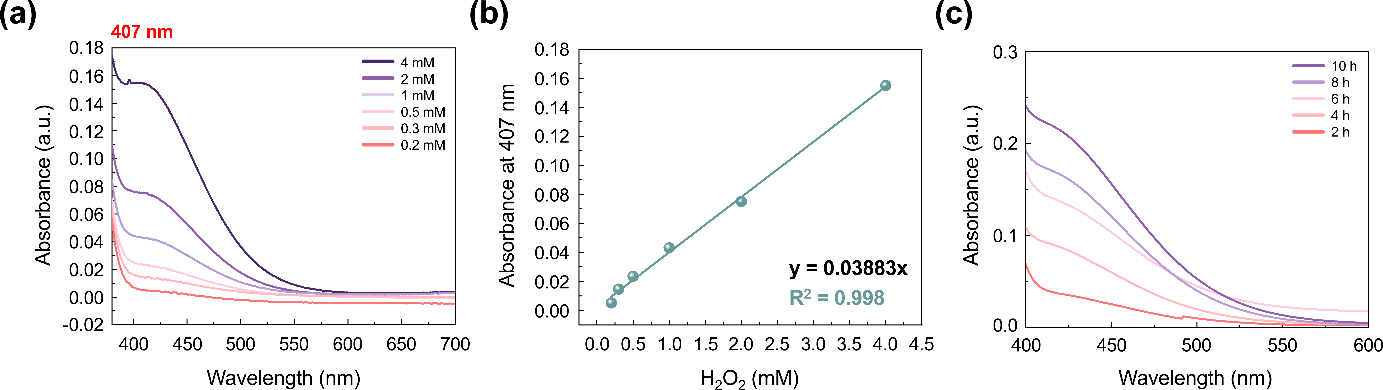
**

**Figure S13.** Colorimetric quantification of H_2_O_2_. The redox colorimetric test for H_2_O_2_ was calibrated by treatment of a series of hydrogen peroxide solutions (concentrations ranging 0.01 to 4 mM) with acidified titanium sulfate solution. (a) A series of H_2_TiO_4_ standards for calibration at 407nm using UV-Visible Spectrophotometers. (b) Calibration curve for the titanium sulfate redox colorimetry test for hydrogen peroxide. (c) A redox colorimetric test for hydrogen peroxide was conducted on MoP Pillar over 28 h at 0.1 V.


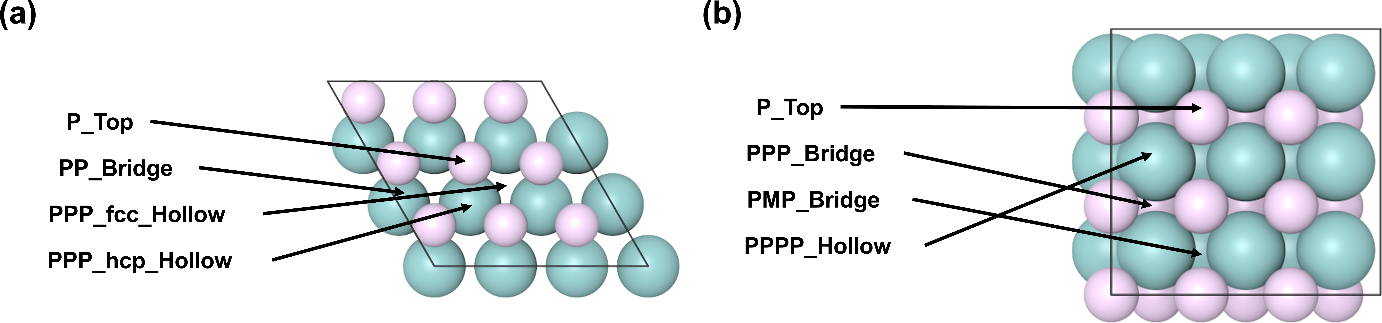


**Figure S14.** Possible adsorption sites on MoP surfaces: (a) Nanoplate and (b) Pillar


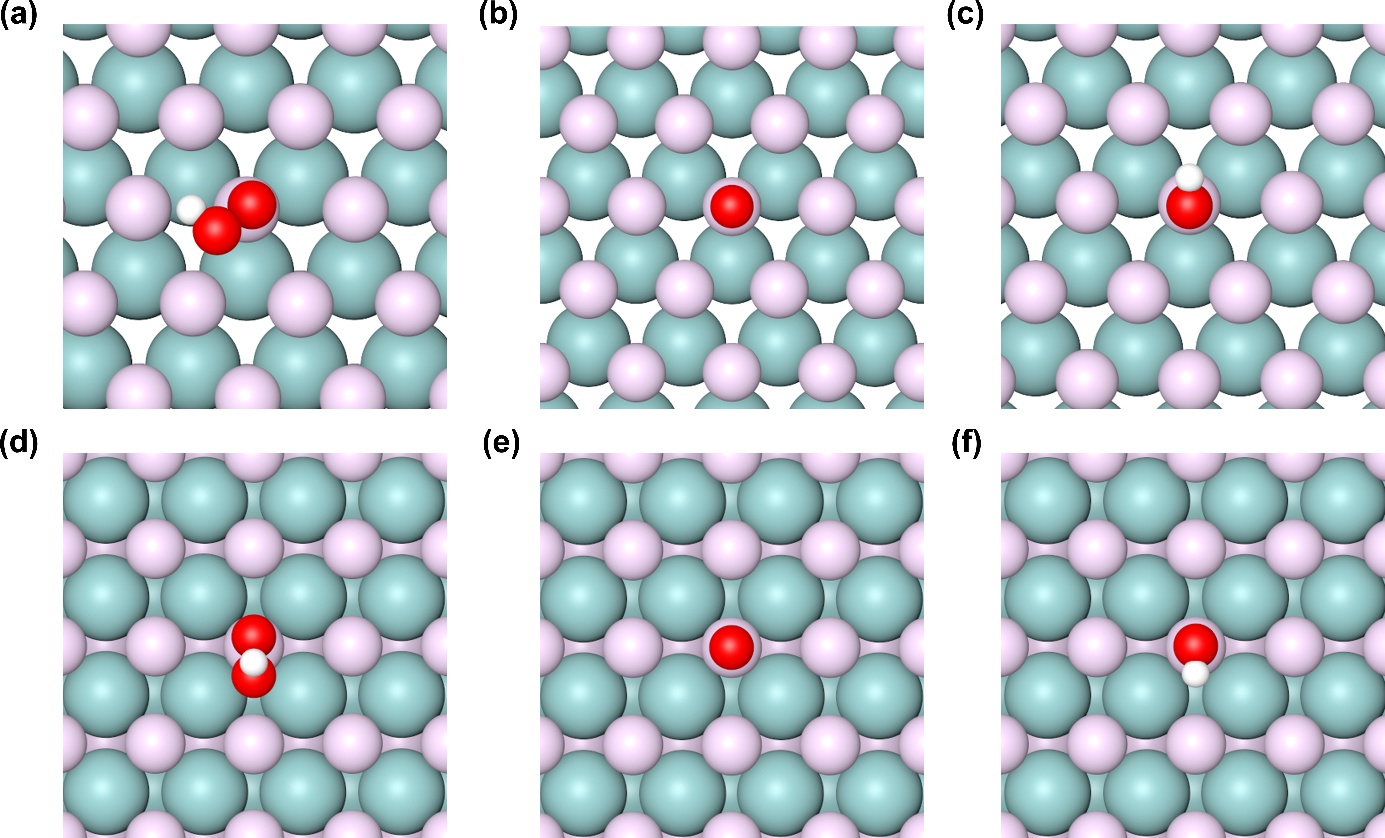


**Figure S15.** Top view of OOH, O, and OH adsorbed on MoP surfaces: (a) OOH, (b) O, and (c) OH adsorbed on $(0001)$ P-terminated nanoplate. (d) OOH , (e) O, and (f) OH on $(10\bar{1}0)$ P-terminated MoP pillar.


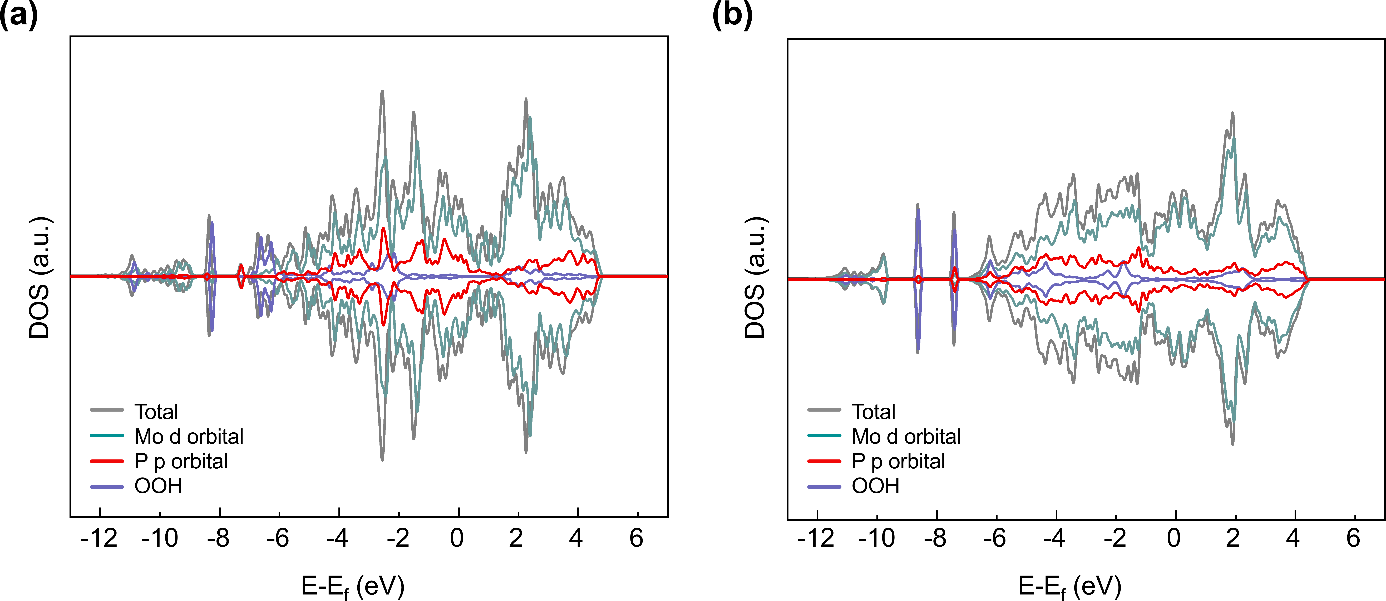


**Figure S16.** Projected density of states (PDOS) plots for MoP surfaces: (a) Nanoplate and (b) Pillar.


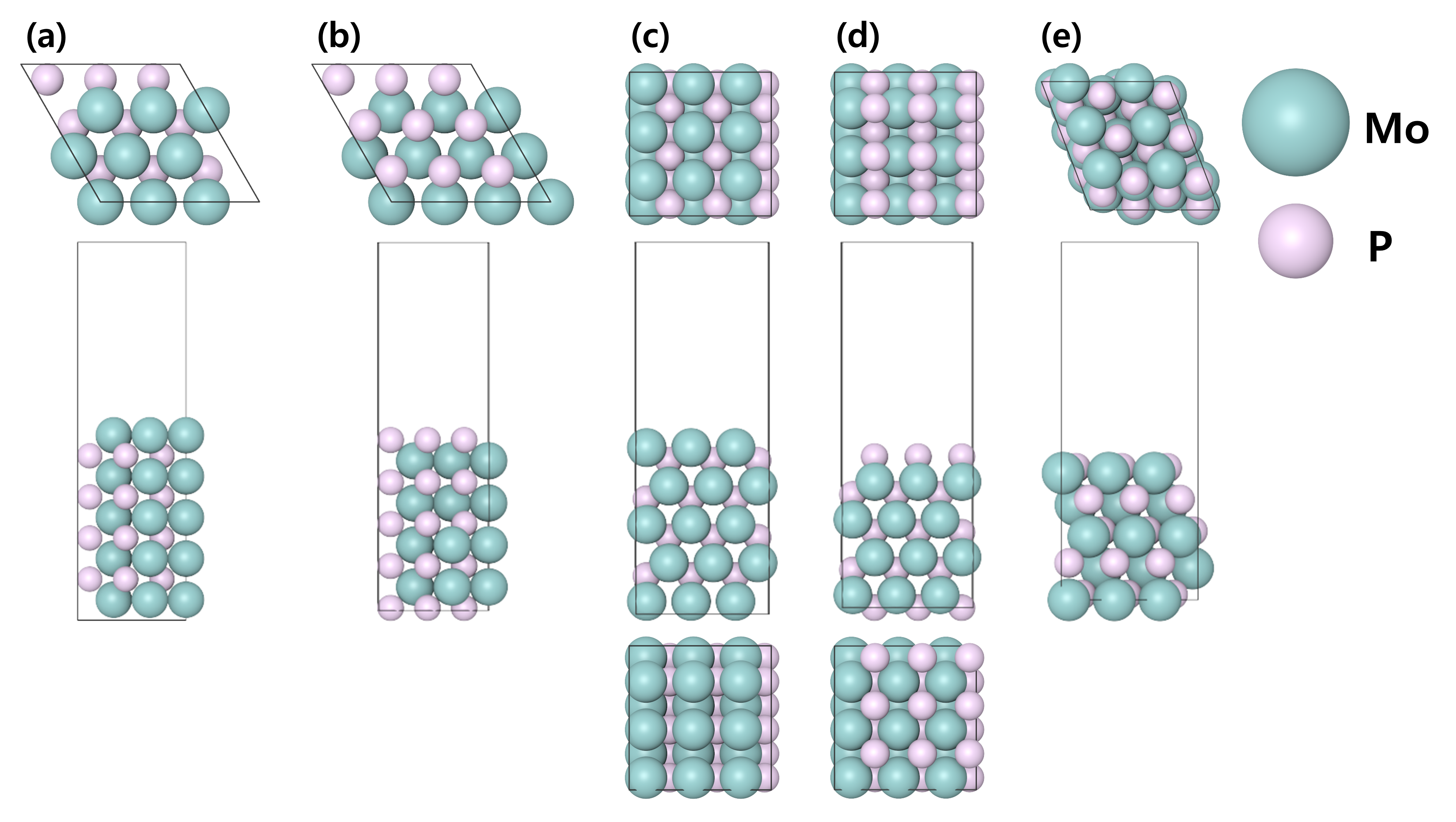


**Figure S17.** Atomic configurations of MoP surfaces: (a) $(0001)$ Mo-terminated surface, (b) $(0001)$ P-terminated surface, (c) $(10\bar{1}0)$ Mo-terminated surface (d) $(10\bar{1}0)$ P-terminated surface (e) $(10\bar{1}1)$ surface.

Table S1. Inductively coupled plasma mass spectrometry (ICP-MS) analysis results comparing the synthesized MoP single crystal with commercial MoP samples.

|  | **Mo**  **(mg/Kg)** | **P**  **(mg/Kg)** | **Ga**  **(mg/Kg)** |
| --- | --- | --- | --- |
| **MoP Single Crystal** | 860213.550 | 145184.744 | 518.166 |
| **Commercial MoP (99.5%)** | 868850.359 | 148386.143 | 16.032 |

Table S2. Comparative analysis of liquid-metal-assisted CVD and conventional CVT methods for crystal growth.

| **Parameter** | **Our CVD Method** | **Traditional CVT** |
| --- | --- | --- |
| Reaction Time | 20 min | 7-14 days |
| Yield per Batch | 20 mg | ~10 crystals (~10 mg) |
| Yield Rate | 60 mg/hr | 0.006-0.012 mg/hr |
| Crystal Size | ~ 150 μm | 500-900 μm |
| Facet | Tunable | Thermodynamically stable facet |
